# Local drivers in accelerating North American heat stress

**DOI:** 10.1038/s41467-026-72795-w

**Published:** 2026-05-19

**Authors:** Andreas F. Prein, Qinqin Kong, Gabriele Villarini, James M. Done, David R. Johnson, Chao Wang, Matthew Huber

**Affiliations:** 1 Institute for Atmospheric and Climate Science, ETH Zürich, Zurich, Switzerland; 2 NSF National Center for Atmospheric Research, Boulder, CO USA; 3 School of Medicine, Stanford University, Stanford, CA USA; 4 Woods Institute for the Environment, Stanford University, Stanford, CA USA; 5 Department of Civil and Environmental Engineering, Princeton University, Princeton, NJ USA; 6 High Meadows Environmental Institute, Princeton University, Princeton, NJ USA; 7 Edwardson School of Industrial Engineering, Purdue University, West Lafayette, IN USA; 8 Department of Political Science, Purdue University, West Lafayette, IN USA; 9 Department of Industrial and Systems Engineering, University of Iowa, Iowa City, IA USA; 10 Department of Earth, Atmospheric, and Planetary Sciences, Purdue University, West Lafayette, IN USA

**Keywords:** Climate change, Natural hazards, Atmospheric science

## Abstract

Climate change increases heat extremes, threatening human health and economies. Using reanalysis data and climate simulations, we show that since the 1940s, population exposure to extreme heat (wet-bulb globe temperature  > 32 °C) has increased by 21% in the U.S. At 2 °C of global warming, exposure increases by 273% because heat-stress frequency increases exponentially with warming. Additionally, 2 °C warming leads to increased nighttime heat stress and decreased work capacity, indicating severe health and economic impacts. Heat stress rises fastest in high-latitude areas, while humid regions experience the greatest exposure increases. In northern regions, heatwave frequency increases with warming, whereas in southern regions, events merge into month-long heatwaves. Increasing temperatures and humidity, along with decreasing wind speed, influence regional heat stress, underscoring the need for tailored adaptation strategies. Overall, heat stress exposure is projected to escalate with additional warming, underscoring the need for mitigation and adaptation to protect vulnerable populations.

## Abstract (web summary)

The authors show that heat stress exposure rises faster with each additional fraction of a degree of warming across North America, meaning even small increases in warming can sharply raise dangerous heat, hot nights, and risks to people.

## Introduction

Climate change is causing shifts in weather patterns worldwide, leading to more frequent and intense extreme weather events^1^. Among these, heat extremes pose a significant threat to human health, eco-, and socioeconomic systems^2–7^. Compared to other weather-related extreme events, heat extremes are causing the most fatalities globally^8–10^. Numerous studies have demonstrated that extreme heat events have already become more frequent and intense and will continue to do so as global warming progresses^1,7,11–13^. Regions in the Global South, such as Southeast Asia, Africa, and Central America, will be disproportionately affected^14,15^ with substantial impacts on poverty in vulnerable regions^16^.

Wet-bulb globe temperature (WBGT) accounts for the combined effects of temperature, humidity, wind speed, and radiation^17^, making it an effective tool for assessing human heat stress. Studies have shown that WBGT performs as well as, or better than, other indices in predicting heat stress^18^, evaluating the physiological impact of heat exposure^19^, modeling heat-related health risks, such as heat stroke morbidity^20^, and examining how multiple meteorological factors interact to influence human physical work capacity^21,22^. WBGT is the U.S. standard heat stress index used by the Occupational Safety and Health Administration^23^, and safety thresholds for WBGT exceedances have been well established for military^24^, occupational^25^, and athletic settings^26^. We apply three widely used WBGT thresholds in this study, all originating from the U.S. military. Green flag hours (GFHs) are met when WBGT > 27.8 °C, and mean cautions are issued for unacclimatized individuals. Yellow flag hours (YFHs) are reached when WBGT > 29.4 °C and are associated with a high risk of heat stress, where reduced work intensity and duration are required. Black flag hours (BFHs) correspond to WBGT > 32 °C and mean extreme risk of heat stress, and all non-essential outdoor activities should stop. WBGT flag categories are defined for occupational heat stress and are not time-of-day specific^27^. Warm nights are known to impair sleep and increase health risks even at lower levels of heat stress^28^. We account for this by analyzing exceedance of GFHs during nights, recognizing that developing nighttime-specific WBGT thresholds remains an area for future work.

WBGT also has some limitations, particularly in underestimating heat stress in humid, low-wind conditions^29^, and issues with reflecting the potentially harmful impact of wind in very hot temperatures^21,30^. Despite these drawbacks, WBGT remains the key index for assessing heat-related health risks and guiding policy decisions for adapting to rising temperatures^23^.

Calculating WBGT requires multiple atmospheric variables, including near-surface temperature, humidity, wind speed, surface pressure, and longwave and shortwave radiation^31,32^. Many simplified approaches have been developed to estimate WBGT^33–36^, but they tend to be systematically biased and generally underestimate peak heat stress^32^. This study calculates WBGT using the physically based approach developed by Liljegren et al. (2008)^31^, which is widely considered the most accurate^37–39^.

We apply this method to a set of current and future climate multi-decadal kilometer-scale (km-scale) simulations over North America called CONUS404^40^ (Contiguous United States 4-km, 40-year-long regional climate simulation) to investigate changes in WBGT extremes, threshold exceedance, and reduction in work capacity with global warming. The simulations can explicitly resolve thunderstorms and local phenomena to provide detailed spatial assessments not available in earlier studies^41,42^. They dynamically downscale fifth-generation ECMWF reanalysis (ERA5) data from 1979 to 2022 and use the pseudo-global warming setup for the future experiment (see Methods for details). All necessary variables are available to calculate WBGT at hourly intervals on a 4 km horizontal grid, providing a much more detailed representation of small-scale processes, including land-surface heterogeneity, topography, local winds, and clouds, than previous studies.

We also apply a sensitivity framework to investigate region-specific drivers of WBGT changes, thereby enhancing our physical understanding of the simulated changes and adaptation capabilities. The explicit identification and quantification of these localized drivers enhance the applicability of the findings for targeted adaptation strategies. Furthermore, we derive a functional form for how the population’s exposure to heat stress will increase with further global warming, a detail often overlooked in prior work^42^.

Here we quantify how heat stress across North America evolves with global warming using physically based WBGT estimates derived from ERA5 and convection-permitting CONUS404 simulations. We assess changes in annual WBGT extremes, threshold exceedance, nighttime heat stress, heatwave behavior, work capacity, and population exposure across historical and future warming levels. We further apply a sensitivity framework to diagnose the dominant local meteorological drivers of WBGT increases. Together, these analyses show where heat stress intensifies most strongly, how rapidly exposure rises with warming, and which processes are most relevant for region-specific adaptation.

## Results

### Simulated WBGT extremes in past, current, and future climates

**Fig. 1:**
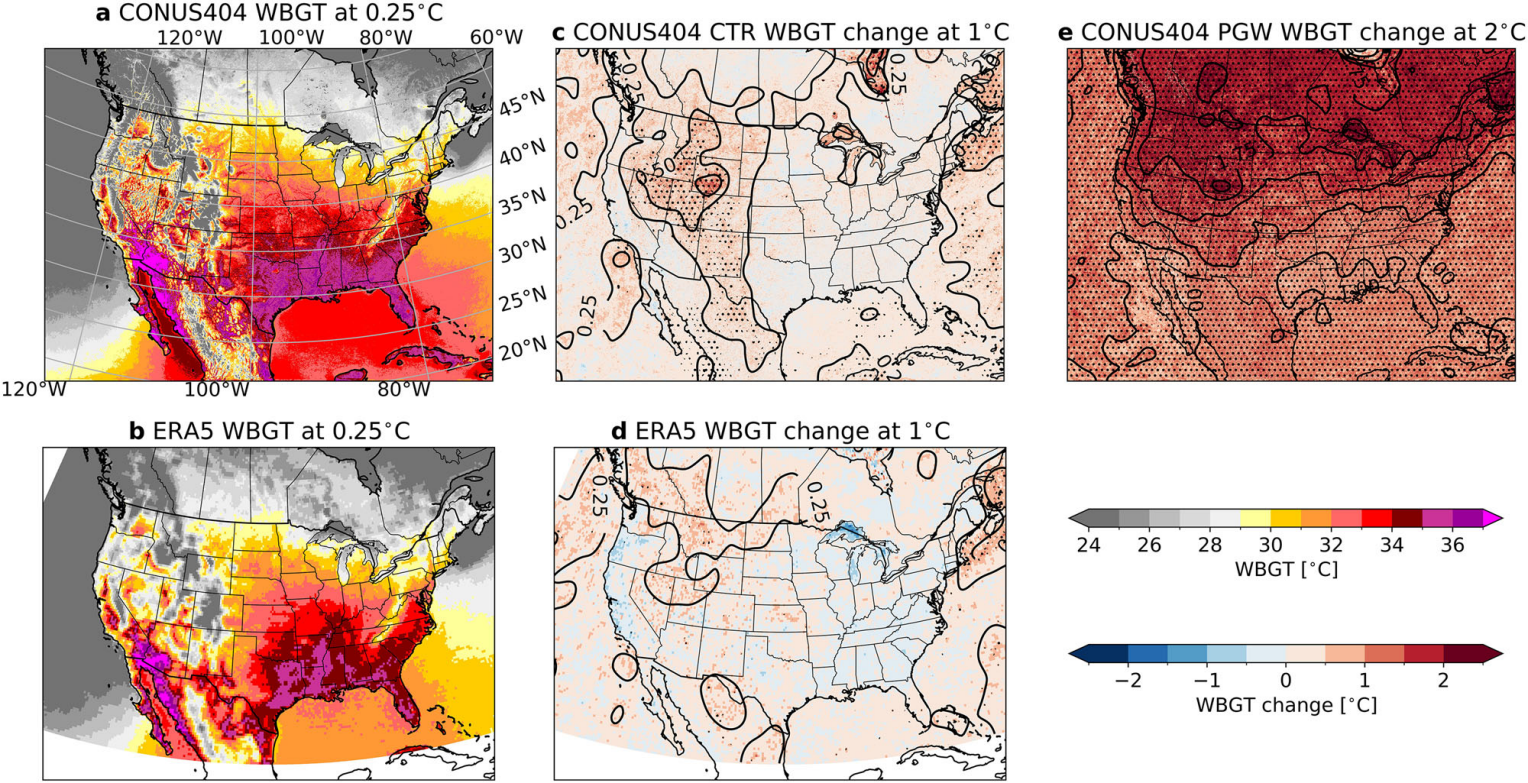
Annual maximum hourly wet bulb globe temperatures (WBGT) increases are the largest in northern land regions and will increase rapidly as we approach 2 °C global warming. Average annual maximum hourly WBGT temperature at 0.25 °C global warming (Fig. 1 **a**, **b**) and its absolute change at 1 °C (Fig. 1**c**, **d**) and 2 °C global warming (Fig. 1**e**) in simulated control (CTR) and pseudo global warming (PGW) CONUS404 (Fig. 1**a**, **c**, **e**) and ERA5 reanalysis (Fig. 1b, d; only 1 °C available). Black contour lines in Fig. 1**c**–**e** show smoothed WBGT isotherms. Hatching in Fig. 1**c**–**e** shows regions with significant changes according to a two-sided Mann-Whitney U test (*p* = 0.05).

The CONUS404- and ERA5-derived average annual maximum hourly WBGT agree well during the baseline period that corresponds to 0.25 °C global warming above 1940–1969 (Fig. 1a, b). However, ERA5 and CONUS404 are not bias-free. Direct evaluation of WBGT is challenging due to the absence of reliable gridded reference data for several required variables. Instead, we evaluate key input variables if reliable reference datasets are available. CONUS404 overestimates surface solar radiation by 10–15% and shows warm biases in near-surface temperature during summer^40^, whereas warm-season wind speed is reasonably well captured^43^. Biases in daily maximum temperature (Tmax) and daily mean dewpoint temperature (TD) on days with annual maximum WBGT are generally  < 2 °C (Supplementary Fig. 1) in most populated regions. The use of a bulk urban scheme in CONUS404 leads to compensating biases of  ~ 1.8 °C in Tmax and  ~ -1.7 °C in TD, caused by underestimated latent and overestimated sensible heat fluxes in cities^44^. We observe anomalously weak heat-stress increases in urban areas relative to suburbs, suggesting that our urban heat-stress projections may be conservative.

All global warming levels in this paper are relative to 1940–1969, which is not the pre-industrial reference period defined in the Paris Agreement^45^. Global mean temperatures during 1940–1969 were approximately 0.2 °C–0.4 °C warmer than mid-19th century pre-industrial conditions, depending on the dataset.

The CONUS404 simulation adds spatial detail to annual maxima WBGT relative to ERA5 by incorporating higher-resolution topography and land-use/land-cover (Fig. 1a, b). Notable differences exist in subtropical ocean regions, where the CONUS404 WBGT is  ~ 2 °C higher. The annual maximum WBGT change at 1 °C global warming is mostly non-significant compared to the baseline period and agrees well between the ERA5 and CONUS404 dataset (Fig. 1c, d) with a tendency of larger increases in CONUS404, especially in the western half of the United States. Larger differences occur over the Great Lakes, where CONUS404 shows an increase in annual maxima WBGT while ERA5 shows a decrease. Note that the higher spatial detail apparent in the annual maxima WBGT in CONUS404 (Fig. 1a) is not apparent in the spatial pattern of the climate change signal. Stark significant increases in annual maxima WBGT appear at 2 °C global warming (Fig. 1e) that are lowest ( ~ 1 °C) in sub-tropical ocean regions and largest ( > 1.5 °C) in northern land regions. We analyze the change in the input variables that are needed to calculate WBGT at the time of the maximum annual WBGT occurrence under 2 °C global warming and find that the changes are associated with significant domain-wide increases in near-surface temperature, specific humidity, and longwave radiation modulated by regional changes in 10 m wind speeds and surface reflected solar radiation changes (Supplementary Fig. 2). Significant changes in the upwelling surface radiation are associated with reduced snow and ice cover in mountainous and high-latitude regions. Annual maximum WBGT increases are not caused by shifts in their seasonal distribution (Supplementary Fig. 2k), since extremes occur at similar times of year in warmer climates compared to the baseline (not shown).

### Heat stress exceedance and work capacity changes with warming

While absolute increases in annual maximum WBGT are largest in northern regions, the societal relevance of these changes depends strongly on background conditions, as regions in the eastern U.S. and along the Gulf Coast already operate close to critical WBGT thresholds.

**Fig. 2:**
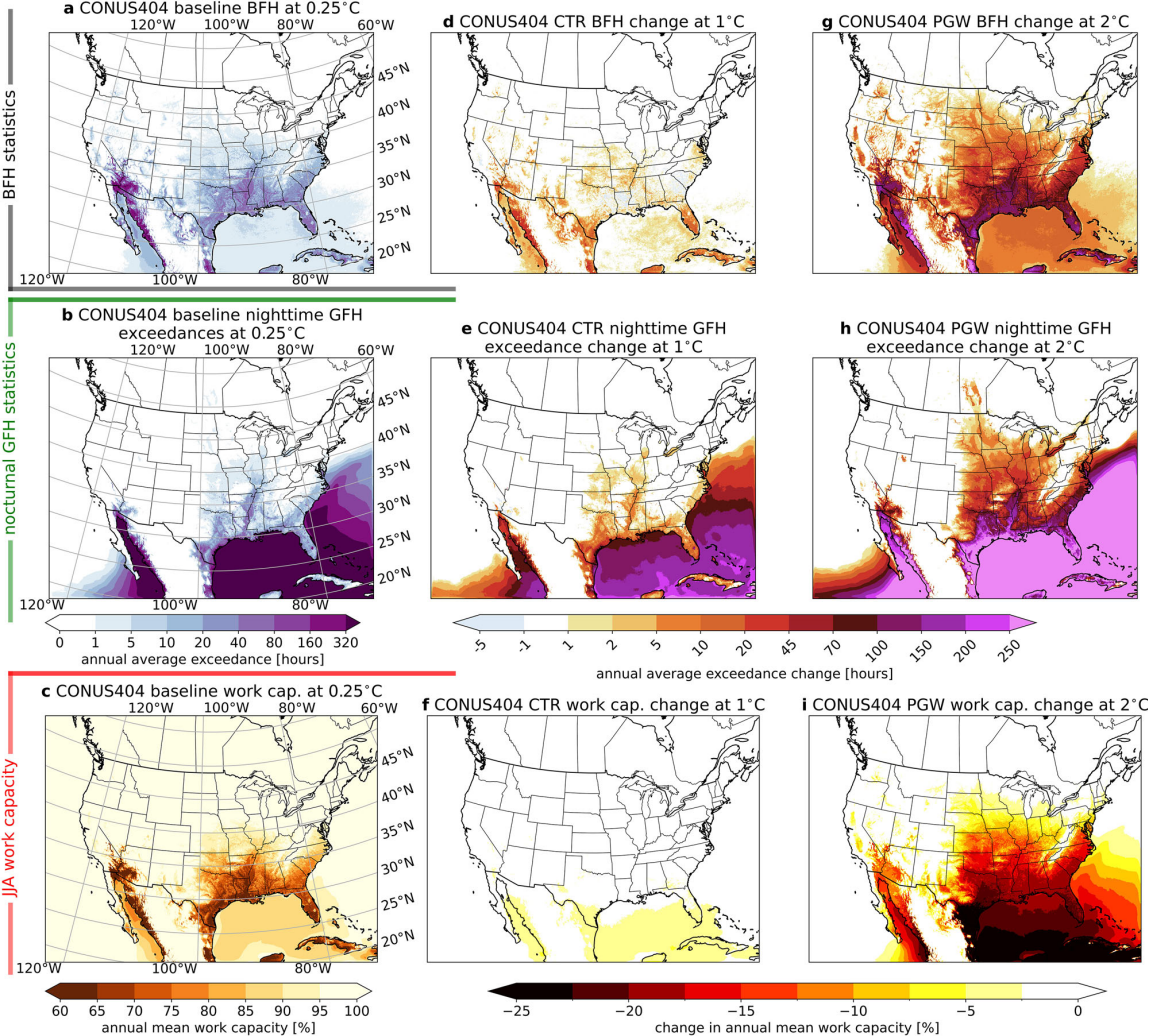
Increases in annual average black flag hours (BFHs), nocturnal green flag hours(GFHs), and decreases in June, July, August (JJA) work capacity are the largest in coastal regions around the Gulf of Mexico, Gulf of California, the U.S. Atlantic Coast, and Caribbean islands with northern regions regularly becoming exposed high heat stress at 2 °C global warming. Panels (Fig. 2**a**, **d**, **g**) show BFH statistics, panels (Fig. 2**b**, **e**, **h**) show nocturnal GFH statistics, and panels (Fig. 2**c**, **f**, **i**) show JJA work capacity. Panels (Fig. 2**a**–**c**) show baseline conditions at 0.25 °C global warming, panels (Fig. 2**d**–**f**) show changes at 1 °C global warming in the control (CTR) simulation, and panels (Fig. 2**g**–**i**) show changes at 2 °C global warming in the pseudo-global-warming (PGW) simulation.

The annual average frequency of BFHs is similar between ERA5 (Supplementary Fig. 3a) and CONUS404 (Fig. 2a) during the baseline period. Both datasets show an exceedance hotspot east and north of the Gulf of California, with secondary hotspots along the Gulf of Mexico coast and the lower Mississippi Valley. These hotspot regions are also the areas where heat stress increased the most in recent decades (i.e., at 1 °C global warming; Fig. 2d, e and Supplementary Fig. 3c, d). Much more pronounced increases occur at 2 °C global warming, with most populated regions in the contiguous United States (CONUS), Mexico, and the Caribbean Islands experiencing substantial increases (Fig. 2g, h). Notably, the strongest increases in WBGT exceedance occur in regions with already high baseline heat stress, particularly the eastern U.S. and the Gulf of Mexico region (Fig. 2). In these areas, even relatively small additional warming leads to disproportionately large increases in exposure because WBGT values are already near or above critical thresholds.

While annual maximum WBGT increases over land are primarily a function of latitude (Fig. 1e and Supplementary Fig. 4a), BFH changes additionally show a strong topographic component with the largest increases at low elevation (with Fig. 2g and Supplementary Fig. 4b). The distribution and increases in YFHs are qualitatively similar to those in BFHs (see Supplementary Fig. 5) but generally much more frequent and widespread, reaching higher latitudes.

Nighttime heat stress has recently been shown as an important contributor to mortality risk under heat extremes^46–48^. The annual maximum daily minimum nighttime WBGT (i.e., the hottest night each year; night being local 20:00–06:00) is increasing faster than the daily maximum WBGT (Supplementary Fig. 3). Nighttime GFHs were rare over land regions during the baseline period and have already increased in the aforementioned heat-prone areas at 1 °C global warming (Fig. 2e). A substantially larger increase is simulated under 2 °C of global warming, particularly in the eastern U.S. and Caribbean regions (Fig. 2h). Especially the heavily populated Gulf and Atlantic Coasts, as well as the southern Mississippi Valley, will see hundreds of hours more nocturnal GFHs per year than under current conditions.

Increasing WBGTs not only threaten human health but also labor productivity. Work capacity (see methods) during June, July, and August (JJA) is already only at 60% in heat-prone regions in the baseline period (Fig. 2c). Only modest changes are modeled at 1 °C global warming (Fig. 2f) while large reductions of up to −25% in southern Texas and northeastern Mexico emerge at 2 °C global warming (Fig. 2i). This indicates a strong nonlinear response to warming, similar to the changes in flag hour exceedance.

**Fig. 3:**
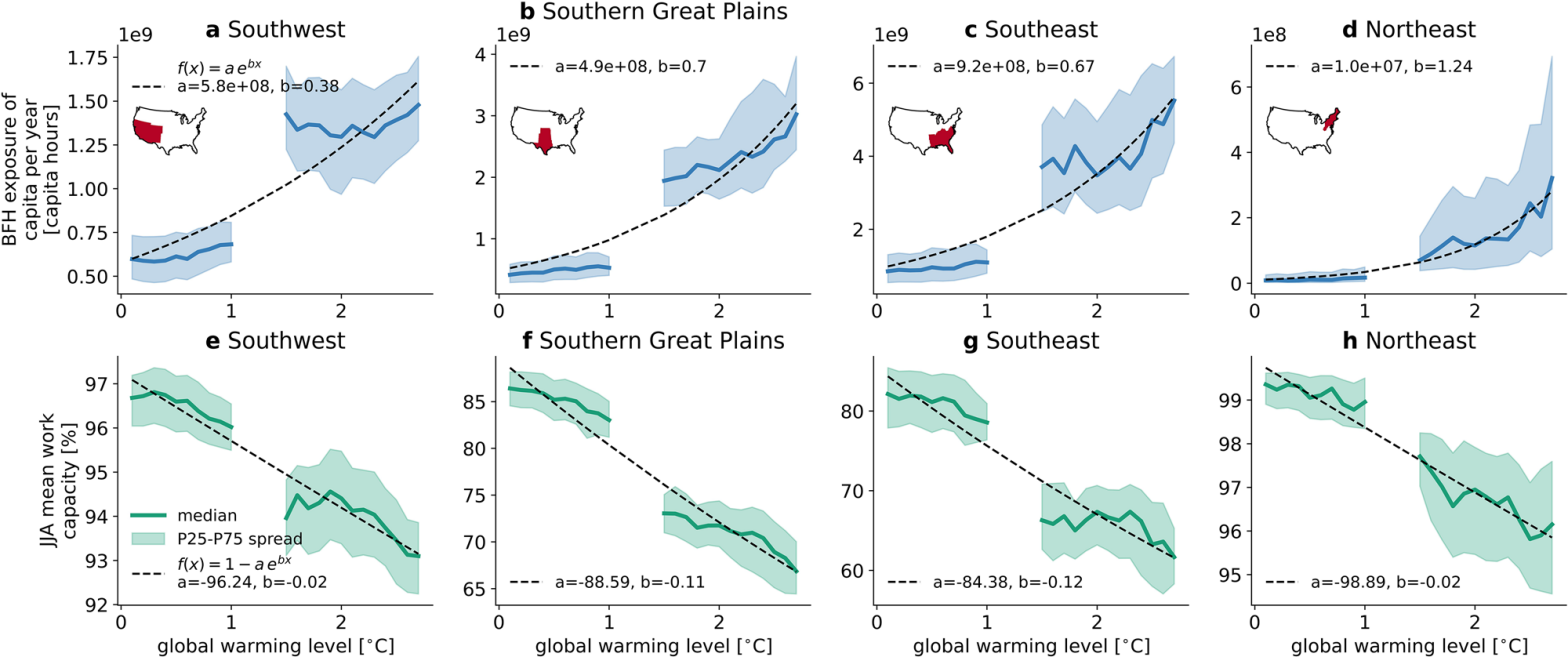
The population exposure to black flag hours (BFHs) is increasing while June, July, August (JJA) work capacity is decreasing exponentially with advanced global warming. Solid blue/green lines (Fig. 3**a**–**d**, **e**–**h**) show the annual median population exposure/summertime median work capacity in selected sub-regions (map inlets). Shading shows the interquartile spread from interannual variability (global warming levels are calculated by sampling over years within a window of  ± 0.25 °C). The dashed line shows the fit of an exponential function (*y* = *a* ⋅ *e*^*bx*^ for BFH exposure and *y* = 1 − *a* ⋅ *e*^*bx*^ for work capacity) where *a* is the initial value at no warming and *b* is the growth rate. The legend shows the best-fit values of *a* and *b*.

The rising WBGTs lead to approximately exponential increases in population exposure to heat extremes (i.e., BFHs) with global warming (Fig. 3a–d, Supplementary Fig. 6; see methods). Historic global warming of 0.25 °C to 1 °C has increased population exposure to BFHs in the contiguous United States (CONUS) by only 21%, which is non-significant in most regions. However, the exponential characteristics of hourly WBGT extremes (Supplementary Fig. 6) result in a much more rapid increase of heat exposure into the future, with a 273% increase of population exposure to BFHs at 2 °C global warming compared to the baseline. Nocturnal GFH population exposure increases more rapidly, two- and ten-fold at 1 °C and 2 °C global warming, respectively, compared to the baseline (Supplementary Fig. 7). Population exposure is highest in the Southeast, followed by the Southern Great Plains and the Southwest for BFH exceedance. In comparison, nocturnal GFH exposure is less severe in the Western U.S. but higher in the Midwest and Northeast. The rate of increase is largest in northern regions, such as the Northeast, where each additional degree of global warming amplifies population exposure by a factor of  ~ 3.5. These increases are approximately exponential, with less good agreement in the southern CONUS. The greater influence of natural climate variability in this region likely contributes to these differences^49^. Additional support for an exponential increase comes from the roughly exponential shape of the right tail in the hourly WBGT probability density functions (Supplementary Fig. 8 and methods). These estimates are likely conservative, as they assume a static population, whereas regions with the highest heat exposure have experienced significant population growth over the last decade^50^. Exponential decreases with warming are found for JJA work capacity changes (Fig. 3e–h). Similar to flag exceedances, absolute changes are largest in the Southern Great Plains and Southeast, while relative change (i.e., decay) rates are largest in Northern regions, such as the Northeast. Levels of 50% work capacity can be expected at warming levels of  ~ 3.5 °C in the Southern Great Plains and Southeast.

**Fig. 4:**
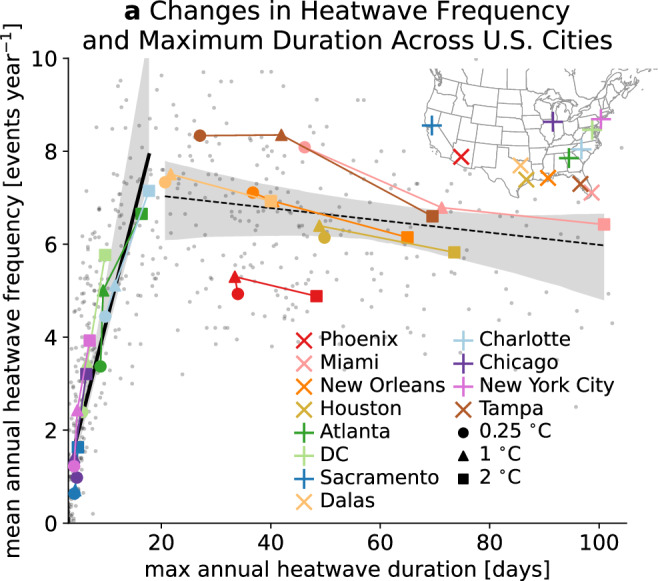
Climate change is increasing heatwave frequency in temperate regions, while in subtropical regions individual heatwaves increasingly merge into prolonged mega-heatwaves. Heatwave frequencies in major U.S. cities increase linearly with lengthening annual maximum heat wave until reaching ~ 7 heatwaves per year (**a**). Afterward, frequencies decrease slowly while the maximum heatwave lengths rapidly increase. Mean annual heatwave frequency compared to maximum annual heatwave duration for 12 major U.S. cities (colors and locations are shown in the inlet). The warming trajectory of each city is shown with a line connecting conditions at three warming levels (0.25 °C, circles; 1 °C, triangles; 2 °C, squares). Thin gray dots show the scatter from all considered years and cities (global warming levels are calculated by sampling over years within a window of  ± 0.25 °C). Darker dots show a higher data density (i.e., multiple points on top of each other). Two trend lines are shown, one for the regime of fast-increasing heatwave frequencies (solid trend line; cities in legend and map that are indicated with plus signs) and a second showing the regime with rapidly increasing max. annual heatwave duration (dashed trend line; cities shown with crosses). Gray shadings show uncertainties in the linear trend fit.

The increases in WBGT will significantly impact the frequency and duration of heatwaves (Fig. 4a). We define a heatwave as a period of at least three consecutive days in which each day includes at least one YFH (we use YFH instead of BFH to increase the statistical sample size). The relationship between annual heatwave frequency and maximum heatwave duration is highly non-linear (Fig. 4a). Cities that experience fewer than seven heatwaves per year experience an increase in heatwave frequency and duration under additional warming. Once a city reaches seven heatwaves per year, frequency decreases slowly, while maximum duration increases rapidly. This shift occurs because individual heatwaves begin to merge into prolonged “mega-heatwaves,” which, in heat-prone cities like Phoenix and Houston, will last longer than a month once global warming reaches 2 °C. This transition–fewer but much longer heatwaves–is not confined to city regions but is widespread across the Gulf of Mexico, the Gulf of California, large parts of Mexico, and the southern U.S. (Supplementary Fig. 9). Moreover, the region experiencing annual heatwaves lasting over 20 days is expanding rapidly as temperatures continue to rise (Supplementary Fig. 9).

Knowing how future warming will affect the frequency and duration of heatwaves is crucial since prolonged exposure to heat extremes heightens the risk of heat-related illnesses^51^, increases energy demand, can damage infrastructure, reduce labor productivity^52,53^, and threaten agricultural productivity and biodiversity^54,55^. Beyond illustrating the heatwave trajectories that urban centers follow under continued warming, Fig. 4a also enables comparisons of heatwave characteristics among cities. For example, under 2 °C global warming, New York City is projected to experience heatwaves as frequent and prolonged as those Atlanta experienced during the 0.25 °C baseline period.

We apply a single set of WBGT thresholds nationwide, which may not reflect regional variations in heat acclimatization. Grundstein et al.^56^ proposed regionally-adjusted WBGT thresholds for athletes over three broad U.S. climate regions. The U.S. military WBGT thresholds used here are broadly consistent with the threshold set proposed by Grundstein et al. for the hottest-humid region. Notably, both the military and athletic thresholds are designed for young, healthy, and physically fit adults. Consequently, the thresholds we use may be overly high for cooler regions and more vulnerable populations, suggesting that our estimates of WBGT exceedance and its temporal changes are likely conservative. Additional insights into heat risk in colder regions can be obtained by considering lower WBGT thresholds corresponding to the yellow or green flags. In fact, the yellow flag threshold lies between the most extreme thresholds defined for the two less hot-humid regions in Grundstein et al. This suggests that WBGT conditions corresponding to the yellow flag in colder regions may represent similar levels of heat risk as the black flag in hot-humid climates. This perspective allows readers to interpret our results in light of regional differences in heat acclimatization.

### Drivers of WBGT increases

**Fig. 5:**
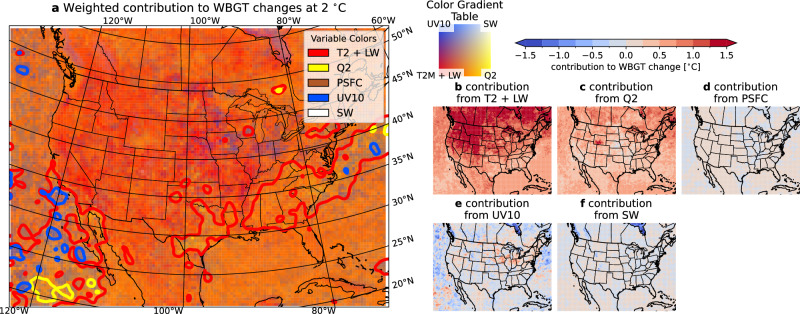
Temperature and longwave radiation increases dominate extreme wet bulb globe temperature (WBGT) changes at 2 °C global warming in most of the study area with important contributions from moisture increases particularly in the South, and wind decreases in the Midwest and Northeast. (Fig. 5**a**) Filled colors show the relative contribution of temperature (T2) and longwave radiation (LW) in red, specific humidity (Q2) in yellow, surface pressure (PSFC) in brown, wind speed (UV10) in blue, and shortwave radiation (SW) in white on the change in annual maxima WBGT extremes. Contours show the area where T2, Q2, or UV10 is the dominant driver of change (i.e., contributing more than 50%). Individual contributions from T2 + LW (Fig. 5**b**), Q2 (Fig. 5**c**), PSFC (Fig. 5**d**), UV10 (Fig. 5**e**), SW (Fig. 5**f**) to annual maxima WBGT changes.

The main drivers of increases in annual maxima WBGT at 2 °C global warming depend on a region’s climate (Fig. 5). Raising air temperatures in combination with increasing longwave heating contribute more than 50% to annual maximum WBGT increases in most regions except for parts of the Gulf of Mexico coastline, the South Atlantic Coast and parts of the subtropical Pacific. The highest temperature and longwave contributions occur in northern land regions and the Rocky Mountains (Fig. 5b). Humidity increases contribute homogeneously across the domain, but their relative contributions peak in humid regions (Fig. 5a, c). Additionally, the U.S. Midwest and the Northeastern U.S. experience increases in maximum WBGT of 0.5 °C to 1 °C from decreased wind speeds during heat events (Fig. 5e). One land region that might see decreases in peak WBGT during heat events due to increasing wind is the coastline of British Columbia. While wind speed changes might be more uncertain in the CONUS404 simulations, the PGW approach can capture systematic large-scale dynamical changes in its forcing data^57^ that contribute to slowing winds during heat extremes.

Locally reduced increases in maximum WBGT are simulated due to reduced outgoing shortwave radiation in high-mountain regions or in water bodies that were historically frozen (Fig. 5f). This is due to a decrease in surface albedo resulting from reduced snow and ice cover. However, this decrease in albedo also increases near-surface temperature (Fig. 5b) due to increased absorption of solar radiation, resulting in a net positive effect on WBGT extremes.

Knowing whether increases in temperature, humidity, radiation, or decreases in wind are most critical in raising heat extremes allows for tailored approaches to mitigating heat extremes^26,58^. When rising air temperature is the key driver, high-albedo materials, green roofs, and strategic shading reduce absorbed heat and surface temperatures in urban settings^59,60^, while choosing heat-tolerant cultivars, improving irrigation efficiency, and maintaining tree canopy reduce thermal stress in agricultural settings^61^. In contrast, elevated humidity calls for ventilation corridors and careful moisture management to improve evaporative cooling and prevent excess fungal growth^62,63^. Where diminished wind flow exacerbates stagnation, urban designs that preserve open spaces and align buildings with prevailing breezes enhance air circulation^64^, and agricultural settings that create wind corridors to enhance airflow can aid pollination and reduce plant heat stress^65^.

While our analysis highlights where temperature-, humidity-, or wind-related factors dominate rising heat stress, a detailed assessment of concrete adaptation options and their economic feasibility is beyond the scope of this work. Nonetheless, identifying the physical drivers provides a foundation for designing region-specific strategies that future interdisciplinary studies can evaluate for cost and effectiveness.

## Discussion

We use state-of-the-art reanalyses and high-resolution climate projections over North America spanning 80 years of historic and future warming^40^ to derive a physically based assessment of extreme heat stress. Using a highly accurate Wet-Bulb Globe Temperature (WBGT)^32^ calculation, we can achieve reliable heat stress assessments at local scales. The results reveal that increases in annual maximum WBGT are spatially smooth and most pronounced in high-latitude land regions, which have historically been considered low-risk. In contrast, the frequency of Black Flag Hours (BFHs; WBGT  > 32 °C) strongly varies regionally, dependent on surface type, topography, and proximity to the coast. This highlights that heat-risk amplification is not driven solely by the magnitude of WBGT change, but critically by pre-existing exposure levels, making humid subtropical regions especially vulnerable even under modest additional warming. BFH frequencies are rising most rapidly in humid subtropical regions, including the highly populated Gulf Coast, South Atlantic Coast, and Caribbean islands. These are also the regions that are most affected by increases in nocturnal heat stress and substantial reductions in work capacity of more than 25% at 2 °C global warming during summer months.

Heatwave impacts on health and the economy depend not only on peak intensity but also on duration and cumulative exposure. Heatwave frequency and maximum annual duration increase linearly with warming until approximately 7 heatwaves per year are reached, beyond which continuous warming leads to the merging of heat events into prolonged mega-heatwaves, persisting for a month when global warming reaches 2 °C in heat-prone regions. Epidemiological studies show that mortality and morbidity rise over successive hot days due to accumulated physiological stress and limited nighttime recovery, with effects often persisting after the event^10,66^. Economic analyses likewise find nonlinear losses in labor productivity and output during prolonged heat, with damages scaling with the number of extreme-heat days rather than single-day peaks^67,68^.

In urban centers, effective adaptation strategies should be region-specific to be most efficient: in humid subtropical regions, humidity is a primary driver of increasing heat stress, while in arid and high-latitude regions, temperature increases are the main concern. Meanwhile, in the Midwest and Northeast, declining wind speeds are an additional factor amplifying heat stress.

While our study is mostly focused on the physical drivers of increasing heat stress, future assessments could use our data to assess heat-stress impacts on economic outcomes and human and ecosystem health, for instance, within the ISIMIP (Inter-Sectoral Impact Model Intercomparison Project) framework^69^.

Population exposure to BFHs is increasing exponentially, exhibiting doubling to tripling rates per degree of global temperature rise in the northern CONUS. While past climate change to date had only minor effects on heat stress in North America, we expect that heat extremes will escalate soon as we approach 2 °C global warming as a consequence of the exponential increase in exposure. While we focus on North America, other regions in the tropics and mid-latitudes are likely to experience similar projections in heat stress. These findings highlight the rapidly evolving risk of heat stress and the necessity for global interdisciplinary efforts^70^ to mitigate climate warming and tailored regional responses to protect human health and livelihood in a warming world^71^.

## Methods

### Datasets

ERA5 is a state-of-the-art atmospheric reanalysis that assimilates observations from surface stations, radiosondes, satellites, and other sources into the European Center for Medium-Range Weather Forecasts’ (ECMWF’s) Integrated Forecast System (IFS), producing global data on a 30 km grid^72^. Recognized as one of the most reliable reanalysis datasets, ERA5 frequently outperforms other products in accuracy^73,74^, though some biases remain^72,73^. For this study, we utilize wet-bulb globe temperature (WBGT) derived from ERA5 instantaneous hourly output and dynamically downscale ERA5, increasing its grid spacing by approximately an order of magnitude.

For the downscaling, the Weather Research and Forecasting (WRF) model^75,76^ was used to refine ERA5 from October 1979 to September 2022 to a  ~ 4 km horizontal grid (hereafter referred to as CONUS404 simulation). The CONUS404 configuration includes 51 vertical levels, with roughly ten levels in the lowest kilometer of the atmosphere. As the successor to the CONUS1 simulation^77^, CONUS404 introduces substantial improvements in representing weather and climate over the central United States^40^, largely due to enhanced land-atmosphere coupling and improved subsurface hydrology^78^. Key CONUS404 settings include the Thompson microphysics scheme^79^, the Yonsei University planetary boundary layer scheme (YSU)^80^, and the Noah-MP Land Surface Model^81,82^ with a 3D groundwater scheme^83^. Urban areas are represented as separate land use types and have specific thermal and radiative properties and roughness (WRF namelist setting sf_urban_physics = 0). This allows for the representation of urban heat islands but results in their overestimation, including too large sensible and too low latent heat fluxes. Land use and land cover settings are kept constant in all WRF simulations using data from 2010. The reduced evaporation in urban areas also results in lower increases in BFH frequencies compared to rural surroundings areas (e.g., see Fig. 2e), an effect that has been found in observations^84^. Radiation processes are handled using the RRTMG shortwave and longwave schemes^85^. To maintain consistency with large-scale atmospheric patterns in ERA5, spectral nudging is applied to CONUS404-s state variables above the planetary boundary layer except for water vapor mixing ratios^86^.

A detailed evaluation of CONUS404’s performance is provided in Rasmussen et al. (2023)^40^. It accurately captures temperature, precipitation, humidity, and radiation patterns but exhibits some biases compared to observational datasets. A cold bias in mountainous regions during winter is linked to limitations in the representation of terrain effects and snow-albedo feedbacks. Precipitation patterns are well-represented, though precipitation amounts are underestimated in the central and southern Plains and in the eastern U.S. during summertime. Near-surface humidity aligns well with gridded observations based on station data, but is overestimated in the southeastern United States during summer. Solar radiation is overestimated, particularly in the Central Plains, likely due to underrepresented shallow cloud cover and aerosol effects. CONUS404 successfully captures long-term regional warming trends and precipitation.

In addition to the historic climate simulation, we also performed a future climate simulation^87^ that uses the pseudo global warming (PGW) approach^88,89^. PGW assumes that the weather of the historic period (1979–2022) reoccurs under future climate conditions by adding monthly mean climatological perturbations to the hourly ERA5 sea surface and lateral boundary conditions. The perturbations are derived from 11-year running average climate change differences by month in the Community Earth System Model version two (CESM2)^90^ second generation 100 member large ensemble^91^. These simulations follow the socio-economic pathway scenario 3 (SSP3-7.0)^92^. For instance, perturbations reflecting the difference between 2025–2035 and 1985–1995 are added to the 1990 ERA5 data to generate sea surface temperature and lateral boundary conditions for 2030. The CONUS404 PGW covers the period from October 2022 to September 2063. Note that the CONUS404 and CONUS404 PGW should not be considered as a continuous integration since the stronger-than-historically observed warming during the last 40 years in CESM2 results in a discontinuity in 2022 that would create an artificially high-temperature trend when considering the simulations as a continuous time series. We use global warming levels as the evaluation framework to mitigate this issue.

Rather than using differences in periods to analyze climate change impacts on future heat extremes, we are using warming levels^93^. Therefore, we calculate global average temperature changes relative to the period 1940–1969 in ERA5 and the CESM2 multi-ensemble mean and focus on three different warming targets. A global warming of 0.25 °C ± 0.25 °C, which occurred during the 1980s and early 1990s (referred to as baseline). A 1 °C ± 0.25 °C warming during the 2010s, and a 2 °C ± 0.25 °C warming that is reached in the 2030s (only available in the CONUS404 PGW simulation). These levels were selected due to their policy relevance and since they are represented in the CONUS404 and CONUS404 PGW simulations.

We used the Gridded Population of the World, Version 4 (GPWv4): Population Density, Revision 11 dataset, developed by the Center for International Earth Science Information Network (CIESIN) at Columbia University. This dataset provides global population density estimates at a high spatial resolution^94^. It represents population counts in 2020 adjusted to administrative boundaries and distributed across a regular grid with a spatial resolution of 5 arc-minutes ( ~ 10 km at the equator).

### Calculation of WBGT

We use Liljegren’s model to calculate WBGT^31^. This approach explicitly simulates energy transfer between WBGT sensors and the ambient environment, incorporating a detailed treatment of sensor geometry and convective, radiative, and evaporative heat transfer processes^31^. It is widely regarded as the most accurate method for WBGT calculation^37^. Liljegren’s model was originally written in Fortran and recently ported to Python with slight adaptations to leverage the full radiation components available in climate model output^32^. This Python implementation is used in our study.

### Assessing the contribution to WBGT changes

Kong and Huber (2025)^95^ recently developed a WBGT sensitivity framework that evaluates the sensitivity of WBGT to variations in temperature, humidity, wind speed, solar radiation, and surface pressure as conceptually illustrated in equation (1).

1 $$\Delta {{{\rm{WBGT}}}}={\gamma }_{T}\Delta {{{\rm{Temperature}}}}+{\gamma }_{Q}\Delta {{{\rm{Humidity}}}}+{\gamma }_{wind}\Delta {{{\rm{Wind}}}}\\+{\gamma }_{SR}\Delta {{{\rm{Solar}}}}\,{{{\rm{Radiation}}}}+{\gamma }_{{P}_{s}}\Delta {{{\rm{Surface}}}}\,{{{\rm{Pressure}}}}$$

In equation (1), the *γ* terms represent the corresponding sensitivity coefficients. Note that the framework is physically based and derived analytically from the heat and mass transfer equations underlying Liljegren’s WBGT model. Please refer to Kong and Huber (2025)^95^ for the derivation of equation (1) and the mathematic forms of the sensitivity coefficients and Δ terms. Note that the Δ*Temperature* term incorporates the effects of changes in longwave radiation, which depends on air temperature.

We apply equation (1) to decompose changes in annual maximum WBGT between the 0.25 °C warming baseline and 2 °C warming into relative contributions of changes in each meteorological input. The Δ terms in equation (1) represent changes between both warming levels conditional on the occurrence of annual maximum WBGT, and can be calculated directly from model output.

However, such decomposition is challenging because the *γ* sensitivity coefficients are not constants and vary across the climatic phase space since WBGT is a nonlinear function of its meteorological inputs. This challenge can be resolved by linearizing the framework by calculating the sensitivity coefficients against certain reference points. In this study, the reference points are selected as local climatology within each grid cell, conditional on the occurrence of annual maximum WBGT across both warming levels. By doing so, the coefficients become constants locally but vary spatially. This choice aims to minimize linearization-induced biases because linearizing against a single location and applying it globally may introduce large biases due to the potentially large spatial variability in the sensitivity coefficients.

We validate the framework by calculating a pseudo change in annual maximum WBGT using equation (1), and compare it against the true values (Supplementary Fig. 10), and found that the spatial pattern of WBGT changes can be well captured by the framework with residual biases mostly within 0.1 °C.

Note that the framework does not assume physical independence among changes in temperature, humidity, wind, and solar radiation. In reality, these variables are dynamically coupled. For example, changes in wind and solar radiation can affect temperature and humidity by influencing circulation patterns and the surface energy balance. Therefore, equation (1) is intended as a diagnostic decomposition of WBGT changes into relative contributions from changes in individual meteorological variables, instead of a disentanglement of the underlying Earth system processes.

### Tail exceedance probability derivation

While the full probability density function (PDF) of hourly WBGT is multi-modal, its right tail features exponential behavior (i.e., close to a straight line on a semi-log plot; Supplementary Fig. 6). The transition from exponential to non-exponential behavior happens at location *x*_0_ (*x*_0_ ~ 30 °C WBGT). We can model this as shown in equation (2).

2 $$f(x)=\left\{\begin{array}{ll}{f}_{{{{\rm{body}}}}}(x),& x < {x}_{0},\\ \pi \lambda {e}^{-\lambda (x-{x}_{0})},& x\ge {x}_{0},\end{array}\right.\\ \,{{\mbox{where}}}\,\,\pi=\int _{{x}_{0}}^{\infty }f(x)\,dx$$

In equation (2)*f*(*x*) describes the entire PDF, *f*_body_(*x*) the body of the PDF except for its right tail, and *π* is the probability mass in the tail.

For a threshold *x*_*t*_ ≥ *x*_0_, the exceedance probability is given by equation (3).

3 $$P(X > {x}_{t})=	 \int _{{x}_{t}}^{\infty }f(x)\,dx\\=\pi 	 \int _{{x}_{t}}^{\infty }\lambda \,{e}^{-\lambda (x-{x}_{0})}\,dx.$$

Making the substitution *u* = *x* − *x*_0_ (so that when *x* = *x*_*t*_, *u* = *x*_*t*_ − *x*_0_) results in equation (4).

4 $$P(X > {x}_{t})=	 \pi \int _{{x}_{t}-{x}_{0}}^{\infty }\lambda \,{e}^{-\lambda u}\,du\\=	 \pi \,{e}^{-\lambda ({x}_{t}-{x}_{0})}.$$

Climate change can be closely approximated by a right shift of the hourly WBGT PDF (see similarity in blue dotted and red lines in Supplementary Fig. 6a, c, e, g). Such a rightward shift of the WBGT PDF is equivalent to lowering the threshold (*xt*) in equations (3) and (4). This shows that the exceedance probability of WBGTs is increasing exponentially if we rightward shift the PDF as long as the exceedance threshold (e.g., BFH; 32 °C) is larger than *x*_0_. Tests with fitting various functions showed that the resulting distribution for the exceedance probability for *x*_*t*_ > *x*_0_ can be best described as exponential or a power-law.

We tested the fit for the same functions to the simulated change in BFH exposure per capita per year (Supplementary Fig. 5). Due to the noisiness of this data and the limited sample size, many functions feature acceptable fits with typical R^2^ of larger than 0.83. Based on this and the above results, we assume that the tail of the WBGT features exponential behavior but acknowledge that there remains some uncertainty.

### Labor capacity function based on ISO/NIOSH workability

To quantify heat-related reductions in outdoor working capacity, we adopt the ISO/NIOSH workability function used in Garcia et al. (2021)^96^ for Europe’s heatwave impact assessment. *WBGT*(*t*) denotes the hourly WBGT [°C] during local working hours (9:00-17:00 local time). The instantaneous work capacity *C*(*t*) ∈ [0, 1] is defined in equation (5) following the ISO/NIOSH heat-exposure formulation.

5 $$C(t)=\max \left(0,\,\min \left\{1,\,\frac{{{{{\rm{WBGT}}}}}_{{{{\rm{lim,rest}}}}}-{{{\rm{WBGT}}}}(t)}{{{{{\rm{WBGT}}}}}_{{{{\rm{lim,rest}}}}}-{{{{\rm{WBGT}}}}}_{{{\rm{lim}}}}}\right\}\right),$$

The thresholds $WBGT_{{{\rm{lim}}}}$ and *WBGT*_lim,rest_ in equation (5) depend on the metabolic workload *M* [W] shown in equation (7).

6 $${{{{\rm{WBGT}}}}}_{{{{\rm{lim,ISO}}}}}=34.9-\frac{M}{46},$$

7 $${{{{\rm{WBGT}}}}}_{{{{\rm{lim,NIOSH}}}}}=56.7-11.5\,{\log }_{10}M,$$

With *M* = {200, 300, 400} in equation (6) and equation (7) represent low, moderate, and high work-intensity tasks, respectively^96^. We set *M* = 300 for our analysis. Thus, *C*(*t*) = 1 implies no heat constraint, whereas *C*(*t*) = 0 indicates heat conditions exceeding the physiological limit for safe work.

We evaluate work capacity across the entire land area and therefore quantify the potential impact of heat on labor capacity rather than the realized economic losses. In reality, only part of the population works outdoors or without air conditioning, and exposure varies by sector and region. A full economic impact assessment would require information on employment structure and cooling availability, which is beyond the scope of this study. Here, we decided to focus on characterizing the physical hazard and the potential constraints it imposes on labor capacity.

## Supplementary information

Supplementary information

Transparent Peer Review file

## Data Availability

The ERA5 reanalysis data used in this study have been deposited in the Copernicus Climate Data Store under the dataset entry *reanalysis-era5-single-levels* https://cds.climate.copernicus.eu/cdsapp#!/dataset/reanalysis-era5-single-levels. The CONUS404 output data used in this study have been deposited in the NCAR Research Data Archive under accession code ds559.0 https://rda.ucar.edu/datasets/ds559.0/. The CONUS404 PGW output data used in this study are available under^87^ https://gdex.ucar.edu/datasets/d559001/. The data that is shown in the article figures can be found in under 10.6084/m9.figshare.31932054^97^.

## Code availability

The code for the statistical analysis and visualization of data in this document can be accessed under https://github.com/andreas-prein/CONUS_WBGT^98^ with the 10.5281/zenodo.15013529.
