# Supplementary material for: Local drivers in accelerating North American heat stress
Source: Nat Commun. 2026 May 19;17:6600. doi: 10.1038/s41467-026-72795-w (PMC13381968; doi:10.1038/s41467-026-72795-w)
Supplement: Supplementary file 1 — Supplementary information [file 41467_2026_72795_MOESM1_ESM.pdf]

Supplementary Information for Local Drivers in Accelerating North American Heat Stress

Andreas F Prein*[1,2], Qinqin Kong[3,4], Gabriele Villarini[5,6], James M. Done[2], David R Johnson[7,8], Chao Wang[9], Matthew Huber[10]

*Corresponding author: Andreas F. Prein, preina@ethz.ch

[1] *Institute for Atmospheric and Climate Science, ETH Zürich, Zurich, Switzerland*

[2] *NSF National Center for Atmospheric Research, Boulder, CO, USA*

[3] *School of Medicine, Stanford University, USA*

[4] *Woods Institute for the Environment, Stanford University, USA*

[5] *Department of Civil and Environmental Engineering, Princeton University, USA*

[6] *High Meadows Environmental Institute, Princeton University, USA*

[7] *Edwardson School of Industrial Engineering, Purdue University, USA*

[8] *Department of Political Science, Purdue University, USA*

[9] *Department of Industrial and Systems Engineering, University of Iowa, USA*

[10] *Department of Earth, Atmospheric, and Planetary Sciences, Purdue University, USA*

Contents

Supplementary Figure 1 3

Supplementary Figure 2 4

Supplementary Figure 3 5

Supplementary Figure 4 6

Supplementary Figure 5 6

Supplementary Figure 6 7

Supplementary Figure 7 8

Supplementary Figure 8 9

Supplementary Figure 9 10

Supplementary Figure 10 11

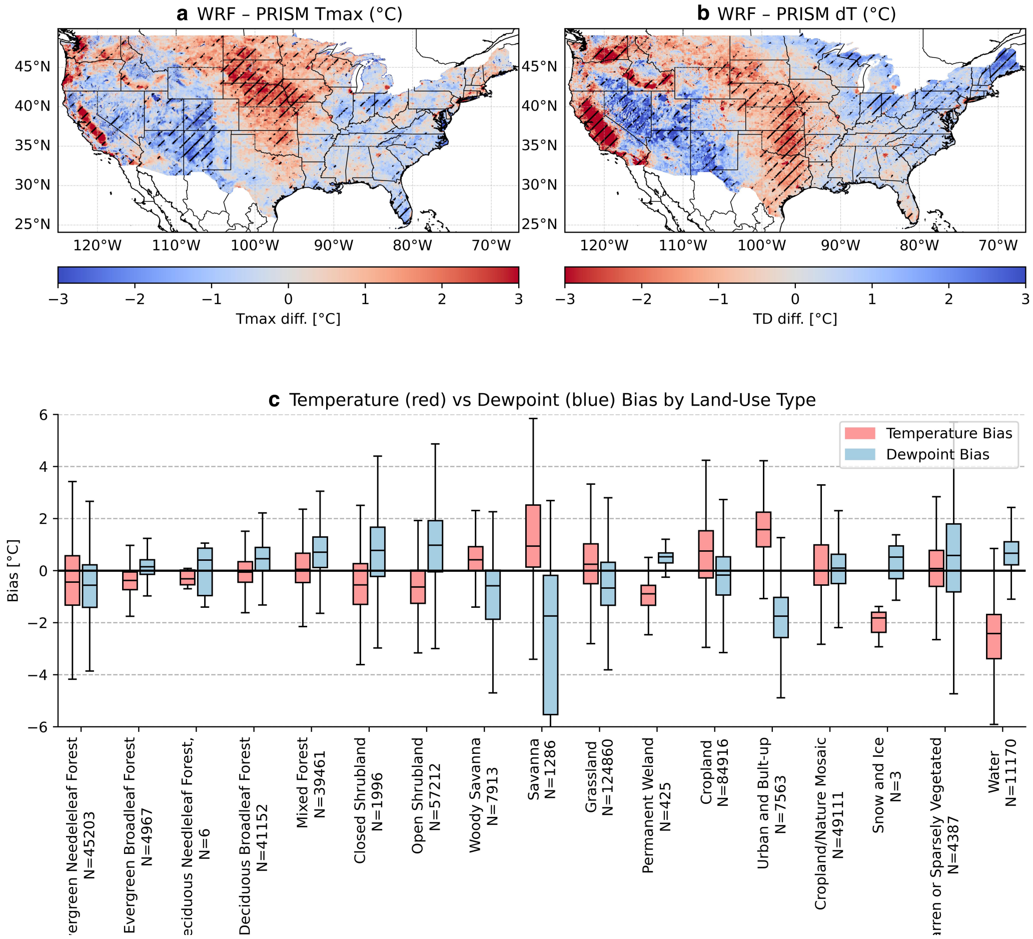

Supplementary Figure 1: **The CONUS404 Weather Research and Forecasting (WRF) simulation has significant warm and dry biases in the High Plains and California Central Valley on days with maximum WBGT temperatures in ERA5.** Average CONUS404 minus PRISM (Parameter-elevation Regressions on Independent Slopes Model) daily maximum temperature (Tmax; a) and daily average dew-point temperature (dT; b) on days with annual maximum wet bulb globe temperature (WBGT) in ERA5. Hatching shows areas with significant year-to-year differences based on a two-sample Student's t-test with autocorrelation-adjusted effective sample size (p=0.05). Box-whisker plots showing annual mean differences in maximum temperature (red) and dewpoint (blue) according to land-use categories in the CONUS404 simulation (c). N denotes the number of grid cells in each category.

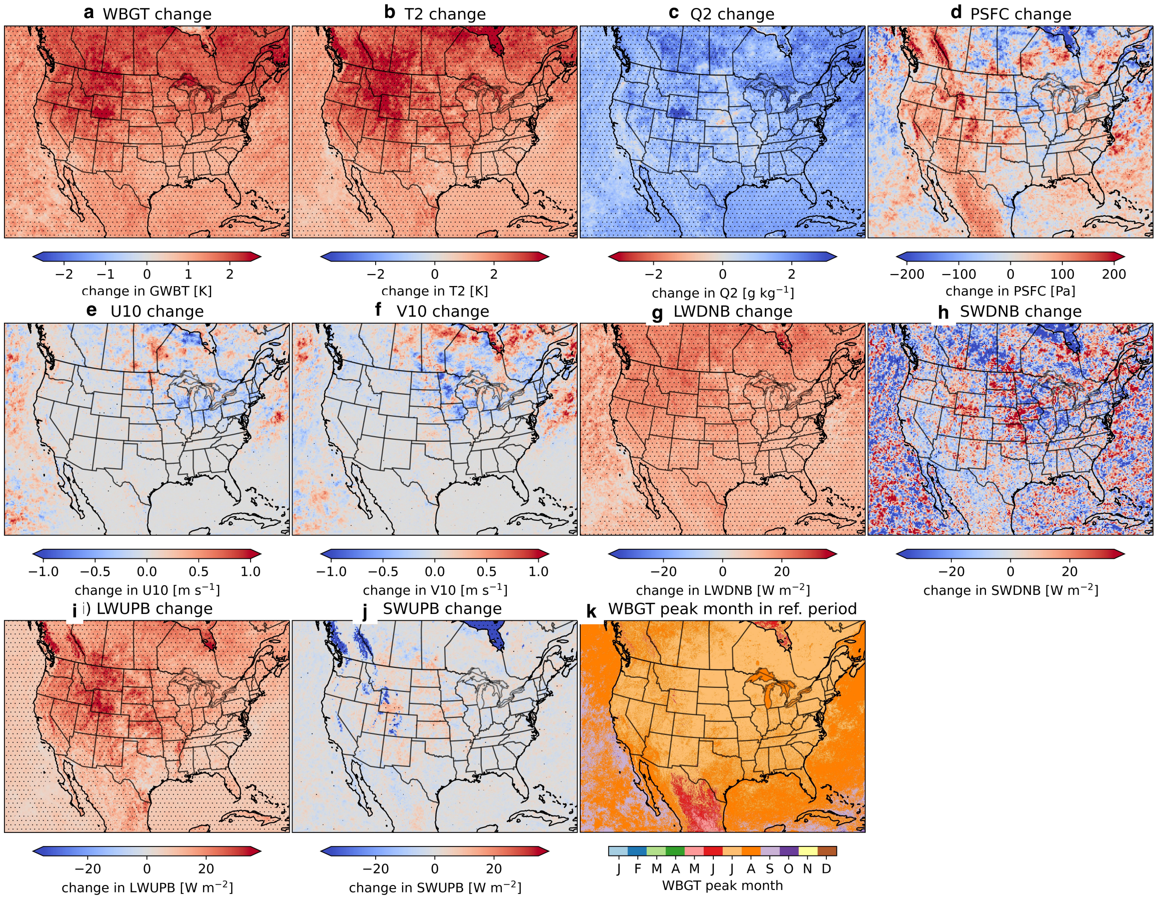

Supplementary Figure 2: **Changes in atmospheric conditions during the occurrence of annual maxima wet bulb globe temperature (WBGT) hours at 2 °C global warming.** Average annual maximum hourly WBGT change (a). Changes in 2 m temperature (b, T2), 2 m specific humidity (c, Q2), sea level pressure (d, PSFC), 10 m zonal (e, U10) and meridional wind (f, V10), downward longwave (g, LWDNB) and shortwave radiation (h, SWDNB) at the surface, upward longwave (i, LWUPB) and shortwave (j, SWUPB) radiation at the surface during the occurrence of annual maximum hourly WBGTs. The median month of WBGT hourly maxima occurrence under the baseline climate (0.25 °C warming; k).

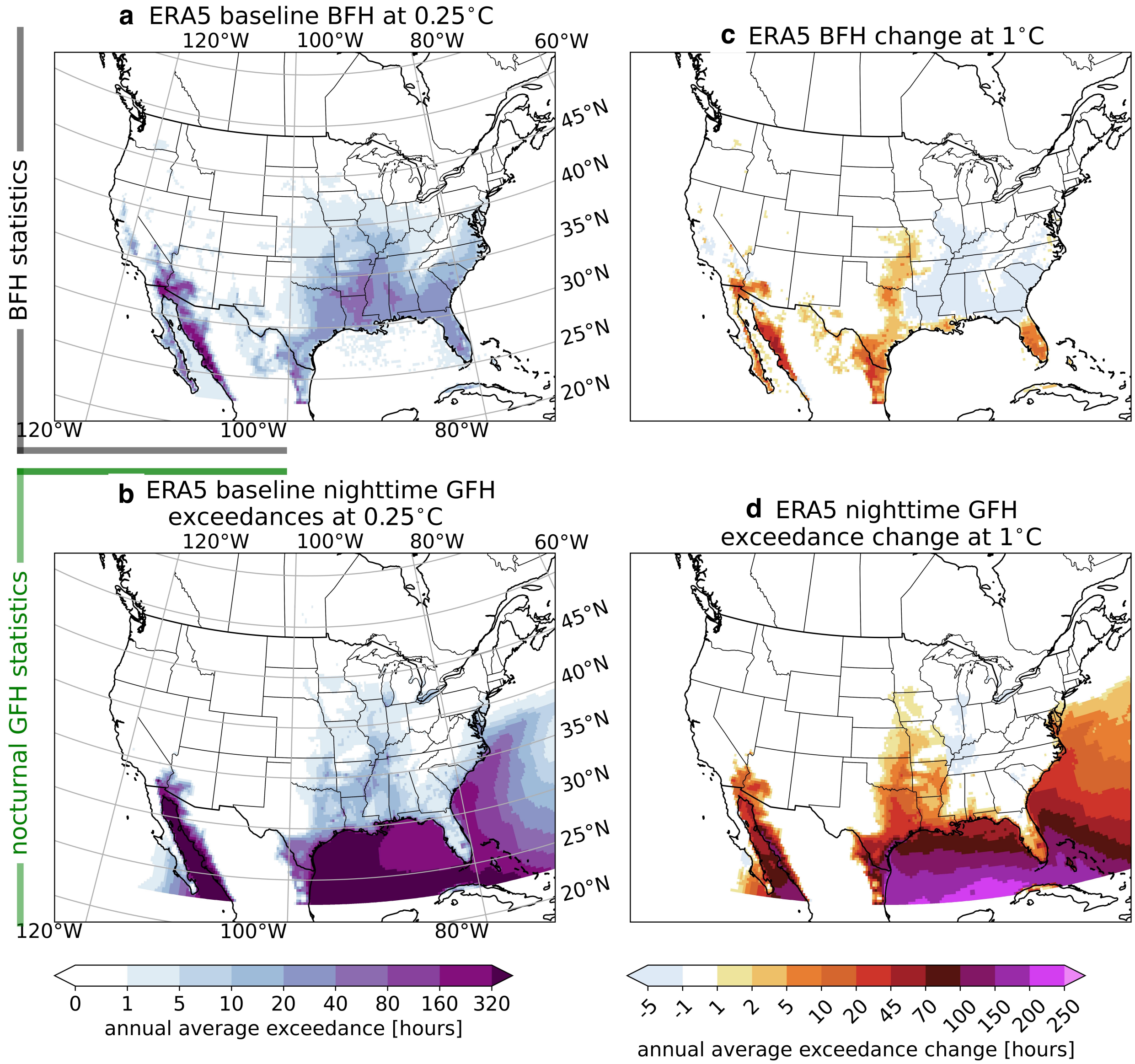

Supplementary Figure 3: **Increases in annual average blag flag hours (BFHs) and nocturnal green flag hours (GFHs) in the ERA5 reanalysis are the largest in coastal regions around the Gulf of Mexico, Gulf of California, the U.S. Atlantic Coast, and Caribbean islands.** Baseline annual average BFH (a) and nocturnal GFH frequency (b) in ERA5 at 0.25 °C. Changes in annual average BFH/nocturnal GFH frequency at 1 °C (c/d) global average warming based on ERA5.

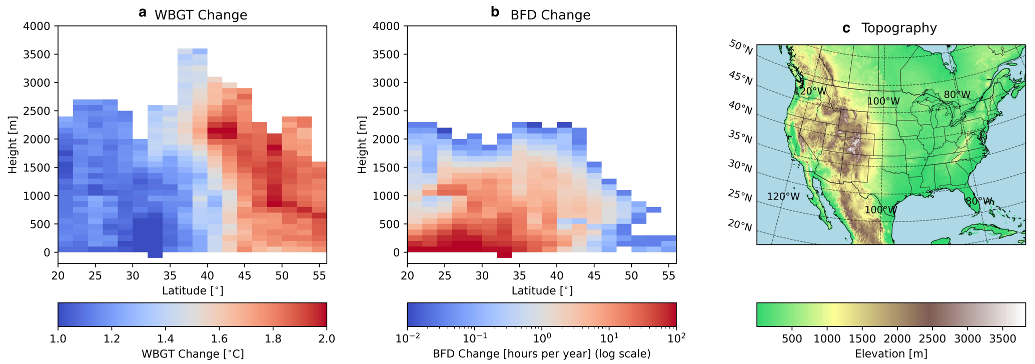

Supplementary Figure 4: **Annual maxima wet bulb temperature (WBGT) changes are primarily a function of latitude, while changes in black flag hours (BFD) exceedances are more dependent on elevation.** Annual maxima WBGT (a) and BFD exceedances (b) dependent on latitude and elevation at 2° of global warming (only land cells are included). The CONUS404 simulation's digital elevation model is shown in (c).

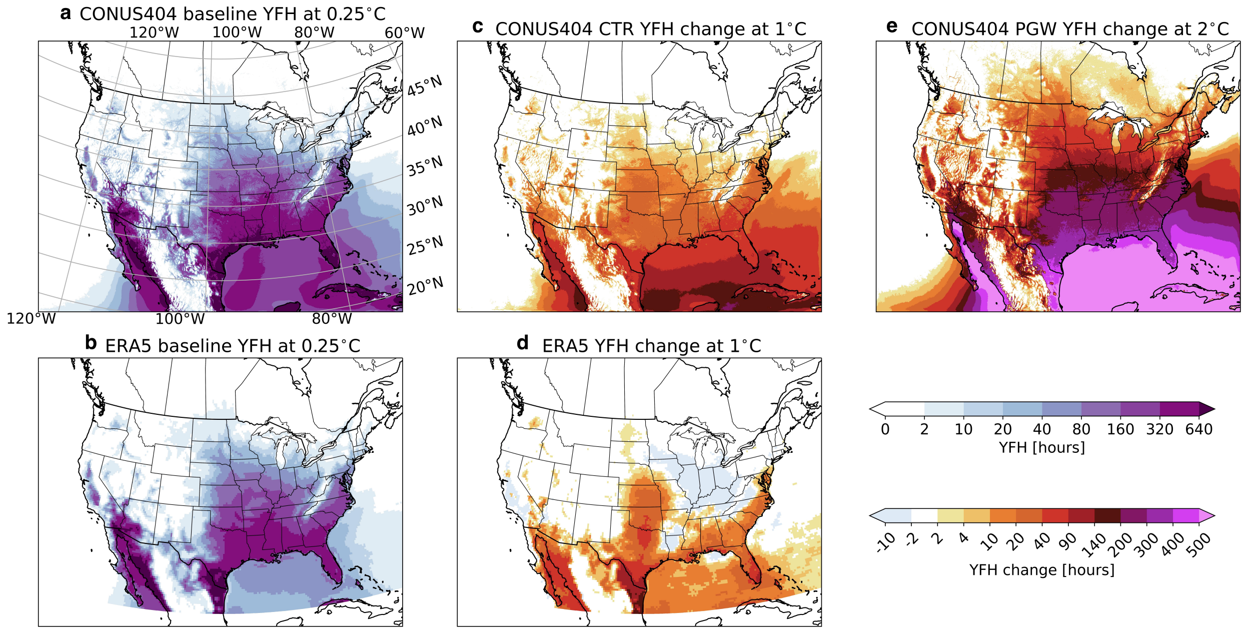

Supplementary Figure 5: **Increases in annual average Yellow flag hours (YFHs) are the largest in coastal regions around the Gulf of Mexico, Gulf of California, the southern U.S. Atlantic Coast, and Caribbean islands with northern regions regularly becoming exposed to YFHs at 2 °C global warming.** Baseline annual average YFH frequency in the CONUS404 simulation (a) and ERA5 reanalysis (b) at 0.25 °C. Changes in annual average YFH frequency at 1 °C (c, CONUS404; d, ERA5), and 2 °C (e, CONUS404) global average warming.

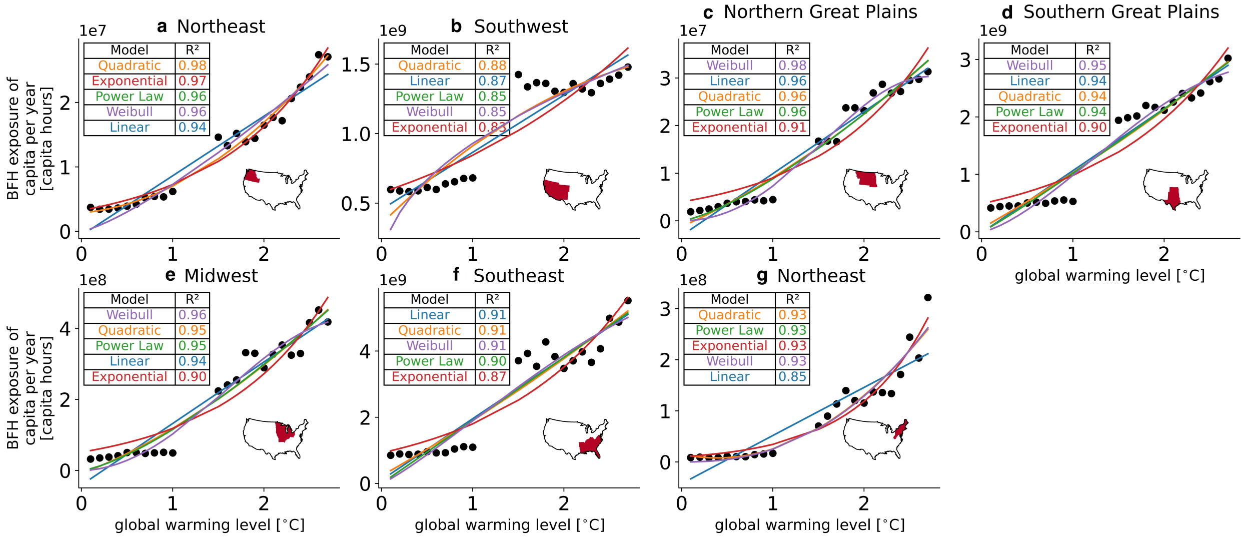

Supplementary Figure 6: **A quadratic function offers the best fit for representing the empirical data of population exposure to black flag hours (BFHs) in most regions of North America**. The black dots show the median population exposure for different global warming levels. Different-colored lines show the best fits of various functions to the empirical data (colors correspond to the font colors in the legend). The coefficient of determination (R^2) is shown for each functional fit (higher is better). Results are shown for the Northeast (a), Southeast (b), Northern Great Plains (c), Southern Great Plains (d), Midwest (e), Southeast (f), and Northeast (g).

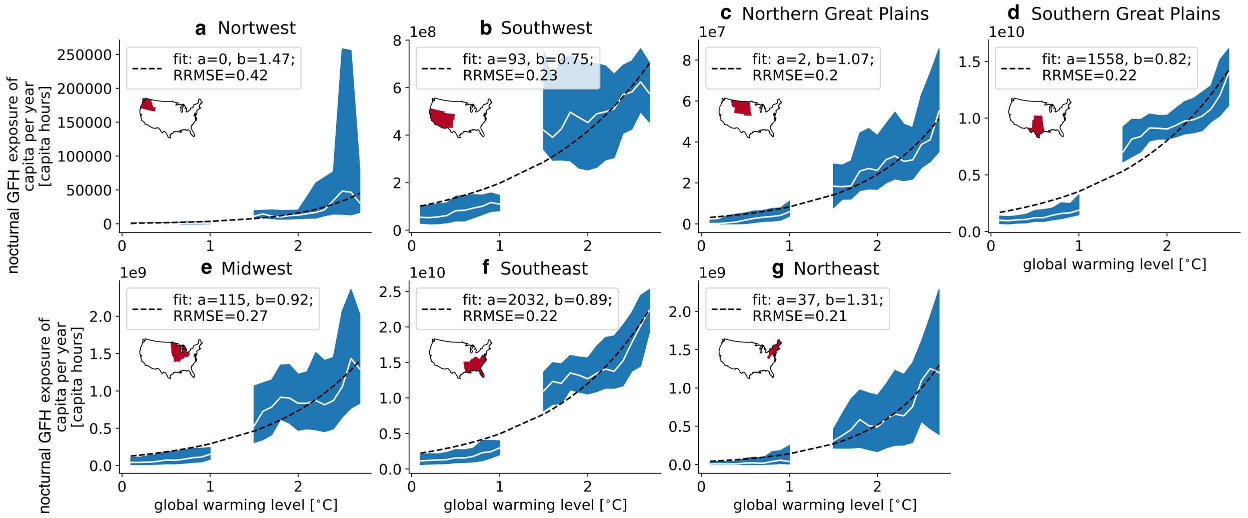

Supplementary Figure 7: **The population exposure to nocturnal green flag hours (GFHs) is increasing exponentially with advanced global warming, with the largest relative increases in northern regions.**. The white line shows the median population exposure, and the blue polygon shows the interquartile spread from interannual variability (global warming levels are calculated by sampling over years within a window of ±0.25 °C). The red polygons in the inlet maps show the seven analysis regions. The dashed line shows the fit of an exponential function ($y = a \cdot e^{bx}$) where a is the initial value at no warming and b is the growth rate. The legend shows the best-fit values for a and b, as well as the relative root-mean-squared error (RRMSE) of the fit. Results are shown for the Northeast (a), Southeast (b), Northern Great Plains (c), Southern Great Plains (d), Midwest (e), Southeast (f), and Northeast (g).

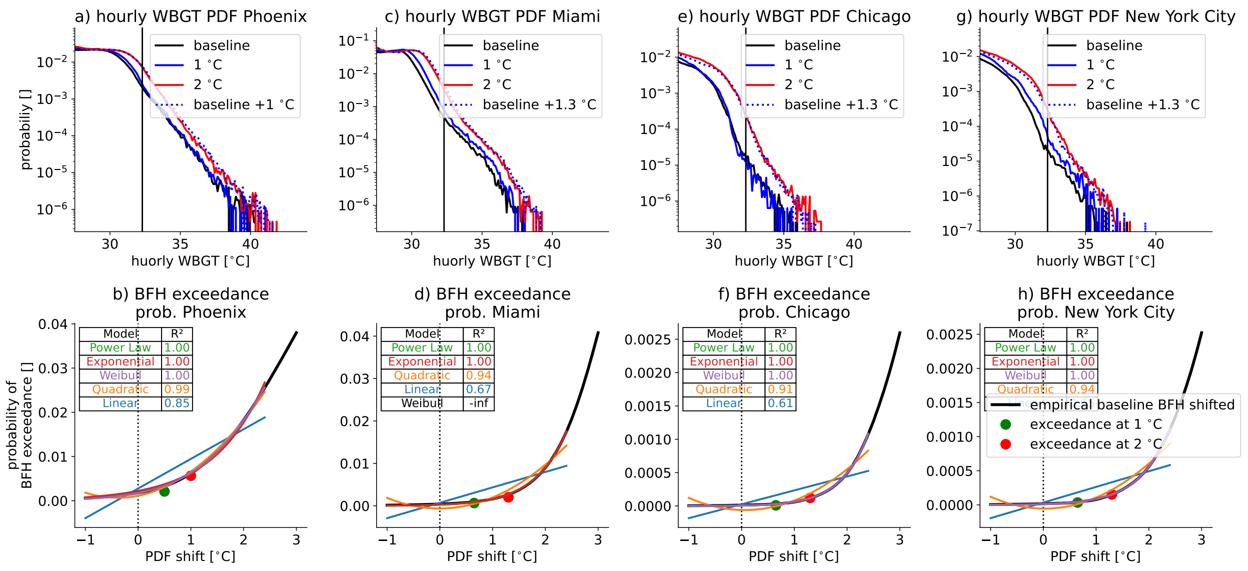

Supplementary Figure 8: **The change in the hourly wet bulb temperature (WBGT) probability density function (PDF) with global warming can be very well approximated by rightward shifting the baseline PDF. The black flag hour (BFH) exceedance probability, thereby, increases approximately exponentially between -1 °C – 2 °C local warming.** Hourly WBGT PDFs for Phoenix (a), Miami (c), Chicago (e), and New York City (g) under the baseline (black), 1 °C (blue), and 2 °C (red) global warming. The blue-dotted PFD is the baseline PDF shifted by 1 °C local warming in Phoenix and 1.3 °C in the other cities. The lower row shows BFH exceedance probabilities (black line) when shifting the baseline PDF to colder or warmer conditions for data from Phoenix (b), Miami (d), Chicago (f), and New York City (h). Different-colored lines show the best fit of various functions to this data (colors correspond to font colors in the legend) between -1 °C – 2 °C. The coefficient of determination (R^2) is shown for each functional fit in the inset table (higher is better). The green/red dot shows the empirical BFH exceedance probability at 1 °C/2 °C global warming derived from the CONUS404 CTR/PGW data.

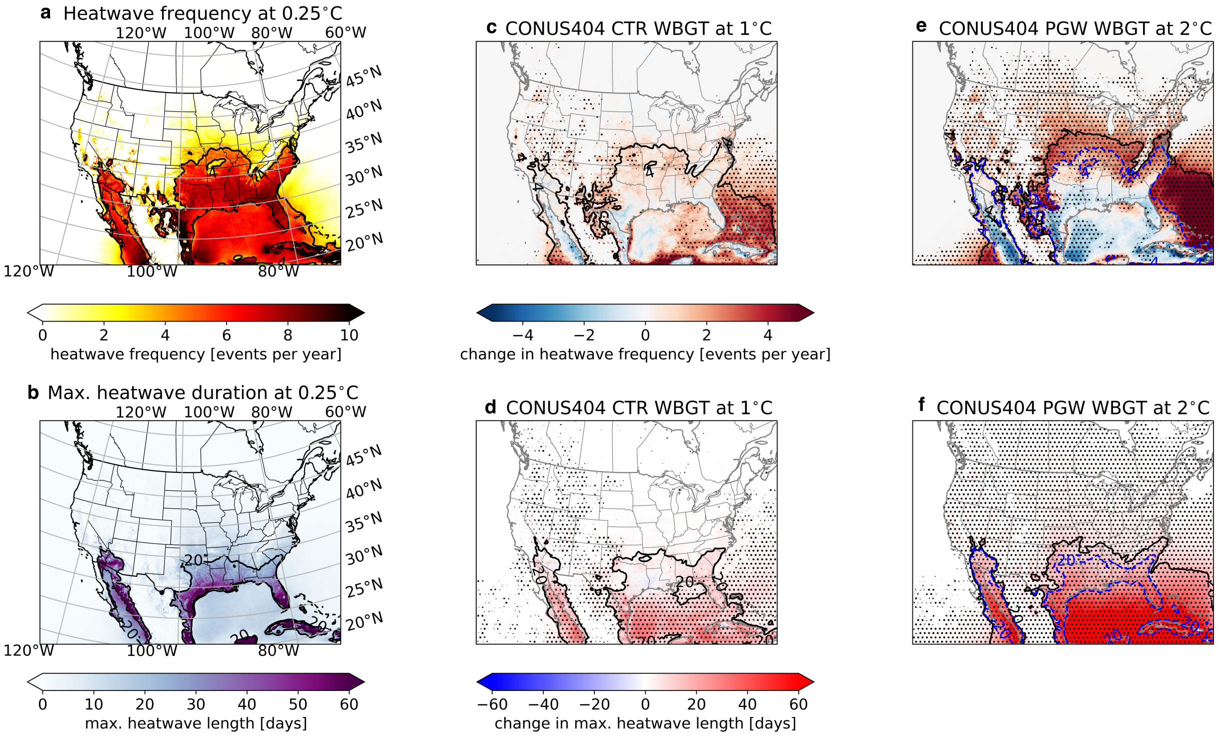

Supplementary Figure 9: **Heatwave frequencies are increasing in the north but decreasing in the Gulf of Mexico and California. The latter is caused by merging of individual heatwaves into mega heatwaves that can last for several months under 2 °C global warming.** Annual heatwave frequencies in the baseline period (a; 0.25 °C global warming) and heatwave frequency changes under 1 °C (b), and 2 °C (c) global warming. Similarly, the average annual maximum heatwave duration and its changes are shown for the same warming levels in panels b, d, and f. Stippling in panels c–f shows significant changes according to a two-sided Mann-Whitney U test (p=0.05). The seven-day heatwave frequency contour is shown as a black line in the top panels. The 20-day average annual maximum heatwave length is shown as a black contour line in the lower panels. The baseline contour line is shown in the 2 °C global warming panels as a dashed blue contour line.

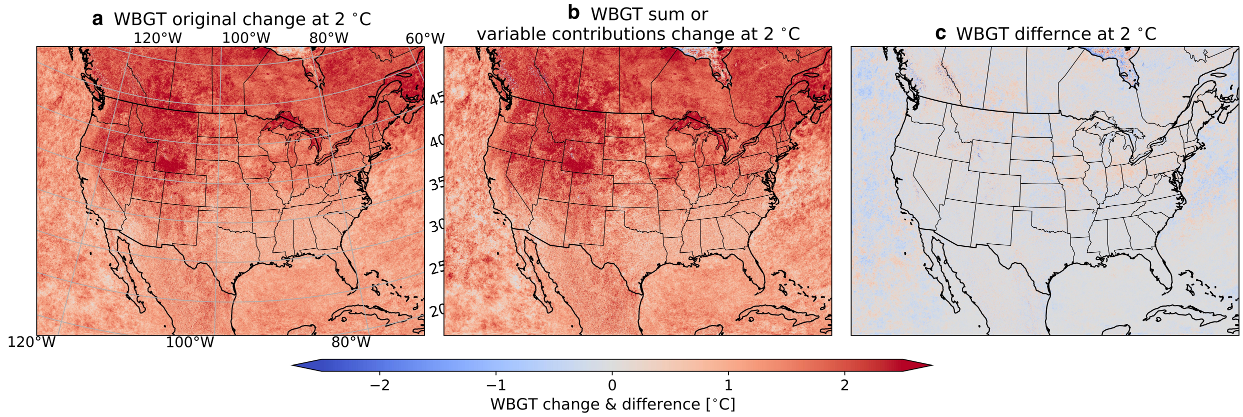

Supplementary Figure 10: **The linearized model to decomposite annual maximum wet bulb globe temperature (WBGT) changes is very closely replicating the original annual maximum WBGT change at 2 °C global warming**. Original annual maximum hourly WBGT change at 2 °C global warming (a) and the sum of the compositional contribution from single variables (b; Equation 1 and Fig. 5 in the main manuscript). The residual error from the total compositional contribution is mostly smaller than 0.1 °C (c).
