# Supplementary material for: Local drivers in accelerating North American heat stress
Source: Nat Commun. 2026 May 19;17:6600. doi: 10.1038/s41467-026-72795-w (PMC13381968; doi:10.1038/s41467-026-72795-w)
Supplement: Supplementary file 2 — Transparent Peer Review file [file 41467_2026_72795_MOESM2_ESM.pdf]

nature portfolio

Peer Review File

Local Drivers in Accelerating North American Heat Stress

Corresponding Author: Professor Andreas Prein

This file contains all reviewer reports in order by version, followed by all author rebuttals in order by version.

Version 0:

Reviewer comments:

Reviewer #1

(Remarks to the Author)

Thank you for the opportunity to review the manuscript titled “Local Drivers in Accelerating North American Heat Stress” by Prein et al. The paper offers a compelling analysis of heat stress patterns across the United States under varying warming scenarios, utilizing cutting-edge data and climate model simulations.
The manuscript’s key strengths include the originality of its concept, the use of advanced datasets and modelling techniques, and its focus on identifying the drivers of heat stress. By integrating observational data with sophisticated climate model outputs, the authors effectively project future heat stress trends and highlight locally specific contributing factors. The study emphasizes the importance of region-specific adaptation strategies to address heat-related challenges. Given its focus and the broader implications, this research will be valuable to scholars working on climate change and heat stress. The methodology appears sound, and the results are well-supported by the analysis. I recommend this manuscript be accepted for publication, subject to a few minor revisions outlined below:
1. Model simulations and heat stress projections

The authors claim that the CONUS404 simulations offer an improved representation of weather and climate parameters due to their ability to capture enhanced physical features. However, there appears to be an overestimation of the urban heat island effect in the simulations. Given this, how do the authors ensure that the simulation of WBGT extremes is reliable across different land cover types, including urban areas? This question becomes particularly important considering the reported simulation of elevated sensible heat fluxes and suppressed latent heat fluxes—key variables closely linked with land surface temperature extremes. This concern is further supported by Rasmussen et al. (2023), as cited in the manuscript, which notes a significant bias in simulated temperature and humidity patterns, particularly in the southern U.S. during summer. How have the authors addressed such biases in their historical simulations using CONUS404? Have any bias correction techniques been applied to the CONUS404 outputs? The manuscript does not clearly explain this. Although Figure 1 demonstrates some agreement between ERA5 and CONUS404 in the spatial distribution of annual maximum hourly WBGT during the baseline period (1940–1969), this alone may not be sufficient to assert that the downscaled dataset is free from bias. How do the authors validate the reliability and bias-free nature of the simulations for historical, current, and future warming scenarios?

2. The use of WBGT-based Flag Hours

• Lines 44–49: The reference to Green, Yellow, and Black flag hours and their associated WBGT thresholds is helpful for contextualizing heat risk levels. However, it is not clear whether these thresholds are universally applicable across all climatic regions of the United States. The manuscript would benefit from a clarification on this point. Additionally, it would be valuable to discuss the role of human adaptation, particularly acclimatisation, in shaping physiological responses to heat. Given that thermal tolerance and heat-related risk thresholds can vary significantly across populations exposed to different climatic conditions, incorporating this dimension could strengthen the interpretation of the findings.

• Lines 105–107: The statement that “BFH changes are much more affected by topography compared to changes in annual maximum WBGTs (compare Fig. 1e with Fig. 2e)” is an important and potentially insightful observation. However, the manuscript does not provide sufficient evidence or analysis to substantiate this claim.
• I understand that the definitions of flag hours (Green, Yellow, and Black) are based on maximum WBGT values, which generally occur during the hottest hours of the day. However, in this study, the same flag hour thresholds appear to be applied to nighttime WBGT values as well. This raises an important question: Should a different set of thresholds be

considered for nighttime conditions, given that human thermal physiology and exposure risks differ significantly between day and night? I understand that to partially address this issue, the authors used GFH instead of BFH. However, it would be valuable for the authors to clarify whether the use of daytime-derived thresholds for nighttime WBGT is methodologically appropriate and to discuss the potential implications of this approach for interpreting heat stress during nocturnal hours.

3. Interpretation of results

• The results of the study can be interpreted from two important perspectives: (1) the increase in maximum annual hourly WBGT (including nighttime WBGT), and (2) the exposure to elevated WBGT values over time. While the authors emphasize that the magnitude of WBGT increase is more pronounced in the northern U.S. under +1°C and +2°C PGW scenarios (Fig. 1 and Fig. S1), an equally critical insight lies in the spatial pattern of exposure to high WBGT values, particularly in the Eastern U.S. and Gulf of Mexico region. In these regions, WBGT values are already approaching or exceeding critical thresholds, and even relatively small increases under warming scenarios could have disproportionately severe impacts. This aspect of high baseline exposure compounded with warming deserves more attention and could be highlighted as an important finding of the study.
• The increase in nighttime WBGT exposure and its implications for Flag Hour categories is particularly important. This is a significant result with implications for adaptation planning. Given its relevance, it is surprising that this finding is presented only in the supplementary material. I recommend that this be brought into the main text and more prominently discussed.
• In Fig S6, it would be interesting to know the locations of empirical exceedance probability at 1, baseline+1 (or 1.3) and 2 degree PGW. This will help in visualising the goodness of fit for the right-tailed exponential fit model.
• Population exposure to BFH for different US regions is also an interesting finding. I am curious to know whether we have the same pattern for the nighttime WBGT (even if it is for the population exposure to GFHs).

4. Drivers of heat stress

The identification of prominent land drivers across different geographical regions of the U.S. is a particularly interesting part of the discussion. This aspect is important, as the characteristics and drivers of heatwaves can vary significantly across climatological zones. I agree with the authors' conclusion that heat mitigation strategies should be region-specific and tailored to the dominant local drivers of heat stress. I found the discussion in lines 154–182 particularly engaging. However, I have concerns regarding the conceptual framework used to support these insights-specifically, the WBGT sensitivity assessment based on the formulation from Kong and Huber (2025), as presented in Equation 1. This framework appears to assume that there is no collinearity among the meteorological variables (e.g., temperature, humidity, radiation, wind speed), which may not hold true in practice. How do the authors account for the potential influence of multicollinearity on the sensitivity coefficients? Without addressing this, there is a risk that the derived sensitivities may be distorted, leading to misinterpretation of the relative importance of different drivers. A brief discussion on this methodological limitation and any steps taken to mitigate its impact would strengthen the analysis.

(Remarks on code availability)

Reviewer #2

(Remarks to the Author)
This paper examines heat stress and its main drivers using Wet-Bulb Globe temperature (WBGT) as a measure to assess heat stress for human health. The measure is based on the combined effects of temperature, humidity, wind speed and radiation. Using factors in addition to temperature alone provides a better assessment of the true stress for the human body. The paper applies this measure to simulated climate scenarios to analyse WBGTs and threshold exceedance with global warming. Moreover, it identifies localized heat stress drivers. It does so using 80 year of historical and future climate data.

The paper finds that increases in yearly maximum WBGT are mostly found in high-latitude land regions. The frequency of threshold exceedance beyond 32 degrees WBGT (Black Flag Hours) are rising in humid subtropical regions. Moreover, once global warming of 2 degrees Celsius is reached and exceeded, the severity and frequency of heatwaves are likely to increase exponentially and also lead to heatwaves merging into a single prolonged heat wave, posing a high risk for human health and the economy.

Finally, the paper calls for mitigation and adaptation strategies and stresses that they need to be adapted to regional specificities for them to be most effective and efficient: in subtropical regions humidity is the main factor of concern; in arid high-latitude regions it is rather temperature increases and in other regions it is wind speeds that can amplify heat stress.

Comments and suggestions:

- The paper is very clear to understand, also for an economist, but we were expecting more analysis in terms of larger impacts such as socioeconomic outcomes in order for the paper to have more impact and go beyond a descriptive analysis.
- The paper's use of hourly data is a major strength, as this high temporal resolution is crucial for evaluating the effects of WGBT on socio-economic sector (e.g. human health and labour productivity). The dataset also offers strong spatial and temporal coverage, effectively capturing key phenomena such as the lack of nighttime cooling and the persistence of heat.

- To stress further the need for (tailored) adaptation and mitigation strategies the paper could add a section in which the economic risks of heat waves as well as the extreme scenarios if global warming reaches 2 degrees Celsius are explained in more detail. (see for example Current and projected regional economic impacts of heatwaves in Europe | Nature Communications which looks into effects on productivity). For example, even WBGTs at threshold levels below 32 degrees i.e. the black flag hours could already reduce productivity.
- The dataset could be better exploited and possibly combined with the Inter-Sectoral Impact Model Intercomparison Project (ISIMIP) downscaled dataset to examine humid heatwaves' impact on socioeconomic outcomes.
- It would also be important to know what tailored mitigation and or adaptation strategies could look like. As the authors point out, they should differ across regions, with high temperatures mattering more in some regions and humidity more in others. Which tailored measures should be implemented? From an economic point of view, it would be interesting to know whether these measures would be costly or how they could be designed to be most cost-effective.
- Novelty: Regarding the main results depicted in Figure 4, we would like to know whether the authors agree that in a setting with 365 days per year the frequency of heatwaves must decrease with the length of heatwaves per year (with 10 heatwaves of 7 days, this implies already 70 days + at least one day below the BFH threshold to separate two heat events). Is the novelty of the finding that there is a turning point precisely at 7 heatwaves per year? And would this point be different under normal climate conditions? In case this is different, what could be the socioeconomic impact of this specific feature?
- Methodology: how were the drivers of heatwaves identified? Are the drivers mutually independent?
- It is important to explicitly mention both in the text and in the figures that point of comparison for the 1.5 degree or 2 degree warming is 1940-1969 instead of the Paris Agreement's time period 1840.

Minor comments:

- Line 20: define explicitly BFH
- Line 81: remove "hourly"
- Line 189: "black flag hours" instead of "black flack hours"
- Figure 4: y axis should state [days]^-1 which is the proper unit used in physics for frequency (number of events per time period)
- Line 120: 273% increase of population exposure
- Please mask the oceans in all the graphs – very difficult to read otherwise
- Please review the colour scales that have continue colours in separate discrete number of colours, this will help readability of the charts. This is not for all the colour scales but for a few of them.

(Remarks on code availability)

Version 1:

Reviewer comments:

Reviewer #1

(Remarks to the Author)
I thank the Editor for providing me with the opportunity to revisit the authors' responses to the comments I raised during the first review of the manuscript. I find that the authors have made a sincere and commendable effort to address the concerns. In doing so, they have undertaken additional analyses and incorporated further discussion into the revised manuscript. Most of the additional analyses are satisfactory and strengthen the robustness of the findings. Overall, I am satisfied with the responses to my comments and with the additional work carried out by the authors.
My broad responses to the individual points are provided below.
Point 1:
I agree that in the absence of fine temporal resolution data, bias correction is difficult. I commend the authors to do additional analysis of comparing model simulation with observed data at daily scale. am satisfied by the response. The additional analysis that authors did is satisfying.
Point 2:
I agree with the authors that there are no physiologically grounded or empirically validated WBGT thresholds. I am satisfied with the additional clarification provided in lines 197–210. The expanded discussion effectively underscores the importance of considering local and regional thresholds when drawing inferences about WBGT exposure. I appreciate this addition and find the revised discussion satisfactory.
Point 3 and Point 4- I am satisfied with the response and the clarification.

(Remarks on code availability)

Reviewer #2

(Remarks to the Author)
Many thanks for your changes and detailed replies to our points. We now believe the paper to be ready for publication.

(Remarks on code availability)

Open Access This Peer Review File is licensed under a Creative Commons Attribution 4.0 International License, which permits use, sharing, adaptation, distribution and reproduction in any medium or format, as long as you give appropriate credit to the original author(s) and the source, provide a link to the Creative Commons license, and indicate if changes were made.
In cases where reviewers are anonymous, credit should be given to 'Anonymous Referee' and the source.
The images or other third party material in this Peer Review File are included in the article’s Creative Commons license, unless indicated otherwise in a credit line to the material. If material is not included in the article’s Creative Commons license and your intended use is not permitted by statutory regulation or exceeds the permitted use, you will need to obtain permission directly from the copyright holder.
To view a copy of this license, visit https://creativecommons.org/licenses/by/4.0/

Reviewer #1 (Remarks to the Author):

Thank you for the opportunity to review the manuscript titled "Local Drivers in Accelerating North A merican Heat Stress" by Prein et al. The paper offers a compelling analysis of heat stress patterns across the United States under varying warming scenarios, utilizing cutting-edge data and climate model simulations.
The manuscript's key strengths include the originality of its concept, the use of advanced datasets and modelling techniques, and its focus on identifying the drivers of heat stress. By integrating observational data with sophisticated climate model outputs, the authors effectively project future heat stress trends and highlight locally specific contributing factors. The study emphasizes the importance of region-specific adaptation strategies to address heat-related challenges.
Given its focus and the broader implications, this research will be valuable to scholars working on climate change and heat stress. The methodology appears sound, and the results are well-supported by the analysis. I recommend this manuscript be accepted for publication, subject to a few minor revisions outlined below:

We thank the Reviewer for their thoughtful and insightful comments that helped to substantially improve our manuscript.

1. Model simulations and heat stress projections

The authors claim that the CONUS404 simulations offer an improved representation of weather and climate parameters due to their ability to capture enhanced physical features. However, there appears to be an overestimation of the urban heat island effect in the simulations. Given this, how do the authors ensure that the simulation of WBGT extremes is reliable across different land cover types, including urban areas? This question becomes particularly important considering the reported simulation of elevated sensible heat fluxes and suppressed latent heat fluxes—key variables closely linked with land surface temperature extremes. This concern is further supported by Rasmussen et al. (2023), as cited in the manuscript, which notes a significant bias in simulated temperature and humidity patterns, particularly in the southern U.S. during summer. How have the authors addressed such biases in their historical simulations using CONUS404? Have any bias correction techniques been applied to the CONUS404 outputs? The manuscript does not clearly explain this. Although Figure 1 demonstrates some agreement between ERA5 and CONUS404 in the spatial distribution of annual maximum hourly WBGT during the baseline period (1940–1969), this alone may not be sufficient to assert that the downscaled dataset is free from bias. How do the authors validate the reliability and bias-free nature of the simulations for historical, current, and future warming scenarios?

We acknowledge that biases exist in the model output, and no bias correction is applied because there are currently no sufficiently reliable gridded observational products with hourly resolution for all variables required to compute WBGT. Rasmussen et al. (2023) report an overestimation of surface solar radiation by about 10–15% and near-surface temperature biases of roughly 1 °C, increasing to ~2 °C over the High Plains and up to ~3 °C in urban areas. Prein (2023) further shows that mean wind speed is reasonably well represented, although observational constraints and comparison remain challenging.

We performed additional analyses to better understand how daily maximum temperatures (Tmax) and daily mean dewpoint temperatures (dT) on days of annual maximum WBGT occurrences in ERA5 are simulated compared to the PRISM dataset (see new Fig. S1 and below). Overall, these fields are well simulated with biases of less than 3 °C except for the Central Valley in California. Urban areas are visible with positive biases in Tmax (median 1.75 °C) and negative biases in dT (-1.8 °C).
We added the following context to the revised manuscript:

L86-94: **CONUS404 overestimates surface solar radiation by 10–15 % and shows warm biases in near-surface temperature during summer, whereas warm-season wind speed is reasonably well captured. Direct evaluation of WBGT is challenging due to the absence of reliable gridded reference data for several required variables. Instead, we evaluate two key inputs—daily maximum temperature (Tmax) and daily mean dewpoint temperature (dT) on annual maximum WBGT days in ERA5. Biases in most populated regions are generally < 2 °C (Fig. S1). The bulk urban representation in CONUS404 leads to compensating biases of ~1.8 °C in Tmax and ~-1.7 °C in dT, caused by underestimated latent and overestimated sensible heat fluxes in cities. This yields anomalously weak heat-stress increases in urban areas compared to suburbs, implying that our urban heat-stress projections are likely conservative.**

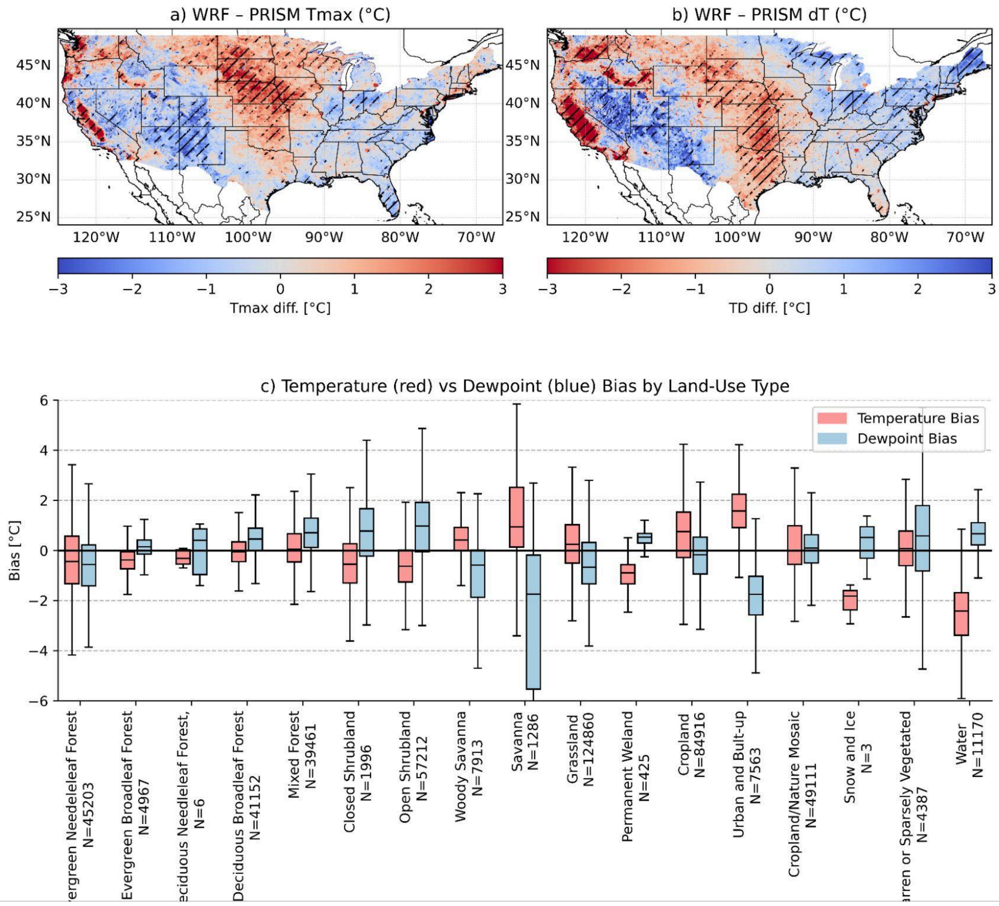

Fig. S1: ***CONUS404 has significant warm and dry biases in the High Plains and California Central Valley on days with maximum WBGT temperatures in ERA5.*** *Average CONUS404 minus PRISM daily maximum temperature (a) and daily average dew-point temperature (b) on days with annual maximum WBGT in ERA5. Hatching shows areas with significant year-to-year differences based on a two-sample Student's t-test with autocorrelation-adjusted effective sample size (p=0.05). Box-whisker plots showing annual mean differences in maximum temperature (red) and dewpoint (blue) according to land-use categories in the CONUS404 simulation (c). N denotes the number of grid cells in each category.*

2. The use of WBGT-based Flag Hours

• Lines 44–49: The reference to Green, Yellow, and Black flag hours and their associated WBGT thresholds is helpful for contextualizing heat risk levels. However, it is not clear whether these thresholds are universally applicable across all climatic regions of the United States. The manuscript would benefit from a clarification on this point. Additionally, it would be valuable to discuss the role of human adaptation, particularly acclimatisation, in shaping physiological responses to heat. Given that thermal tolerance and heat-related risk thresholds can vary significantly across populations exposed to different climatic conditions, incorporating this dimension could strengthen the interpretation of the findings.

Thank you for raising this important point. We agree that heat acclimatisation can enhance heat tolerance through physiological adaptations such as increased sweat capacity, enhanced vasodilation, and improved fluid regulation. Consequently, a single set of WBGT thresholds should not be expected to be universally applicable across all climatic regions of the U.S. However, at present, there are no physiologically grounded or empirically validated WBGT thresholds that vary regionally to explicitly account for differences in acclimatisation. Moreover, acclimatisation itself is dynamic under climate change, potentially implying different thresholds between historical and future periods. Heat tolerance also varies substantially within regions across demographic groups, depending on age, health status, and socioeconomic vulnerability. Taken together, these factors make it challenging to define region-, period-, or population-specific WBGT thresholds in a robust and consistent manner within the scope of this study.

Grundstein et al. (2015) made such an attempt and produced regionally adjusted WBGT thresholds for athletes across three broad regions in the U.S. (Fig. R1; Table R1). Their approach combines the original WBGT thresholds from the American College of Sports Medicine (ACSM) with the 90th percentile of warm-season daily maximum WBGT in each region. While this represents a useful step toward accounting for regional acclimatisation, the resulting thresholds remain statistically-derived rather than supported by physiology or empirical calibration. The choice of 90th percentile is also somewhat arbitrary.

As shown in Table R1, the U.S. military WBGT thresholds used in this study are broadly consistent with the thresholds proposed by Grundstein et al. (2015) for Region 3, which are the highest among the three regional threshold sets defined in that study. Meanwhile, both the U.S. military and ACSM WBGT thresholds are designed for young, healthy, and physically fit adults. Consequently, the thresholds we use may be overly high for cooler regions and more vulnerable populations. In this sense, our estimates of WBGT exceedance and its temporal changes are likely conservative.

However, insight into potential exposure levels in colder regions can be gained by considering lower WBGT thresholds, such as the green and yellow flags. As shown in Table R1, the yellow flag threshold lies between the most extreme thresholds defined for Regions 1 and 2 in Grundstein et al. (2015). This suggests that WBGT conditions corresponding to yellow flag in colder regions may represent a similar level of heat risk as black flag in hot-humid climates. This perspective allows readers to interpret our results in light of regional differences in heat acclimatisation.

We have added a discussion in the revised manuscript (Lines 197–210) to discuss the role of heat acclimatisation and to guide readers in interpreting our results using lower WBGT thresholds for less acclimatised people in colder regions.

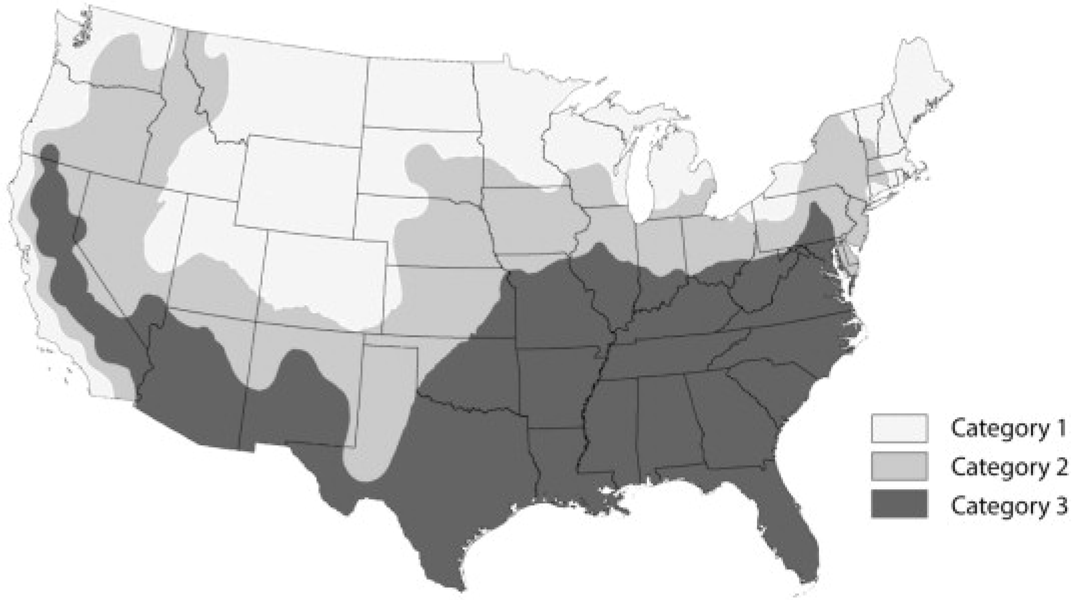

Fig. R1 The three regions defined in Grundstein et al (2015) for determining regionally-adjusted WBGT thresholds. These three regions are determined based on warm-season WBGT climatology.

Table R1 Regionally-adjusted WBGT thresholds for athletes proposed by Grundstein et al (2015). The U.S. military thresholds for Green, Yellow, and Black Flags used in this study are also shown.

**Grundstein et al. thresholds for athletes**			**Activity guidelines**	**U.S. military thresholds**	**Flags**
**Region 1**	**Region 2**	**Region 3**			
24.6-26.7	26.6-28.7	27.9-30.0	Plan intense or prolonged exercise with discretion	27.8	Green
26.8-28.9	28.8-30.9	30.1-32.2	Limit intense exercise and total daily exposure to heat and humidity	29.4	Yellow
>29.0	>31.0	>32.3	Cancel exercise	32	Black

• Lines 105–107: The statement that "BFH changes are much more affected by topography compared to changes in annual maximum WBGTs (compare Fig. 1e with Fig. 2e)" is an important and potentially insightful observation. However, the manuscript does not provide sufficient evidence or analysis to substantiate this claim.

Thank you for raising this point. We added a new figure to the supplement (Fig. S4; see also below) that shows the relationship between annual maximum WBGT changes and BFH exceedances at two degrees of global warming as a function of latitude and elevation. This shows more clearly that the annual maximum WBGT changes are primarily a function of latitude, while BFH exceedances are strongly related to topography (note the logarithmic color table). We added a discussion of these results to the revised document in L133-7.

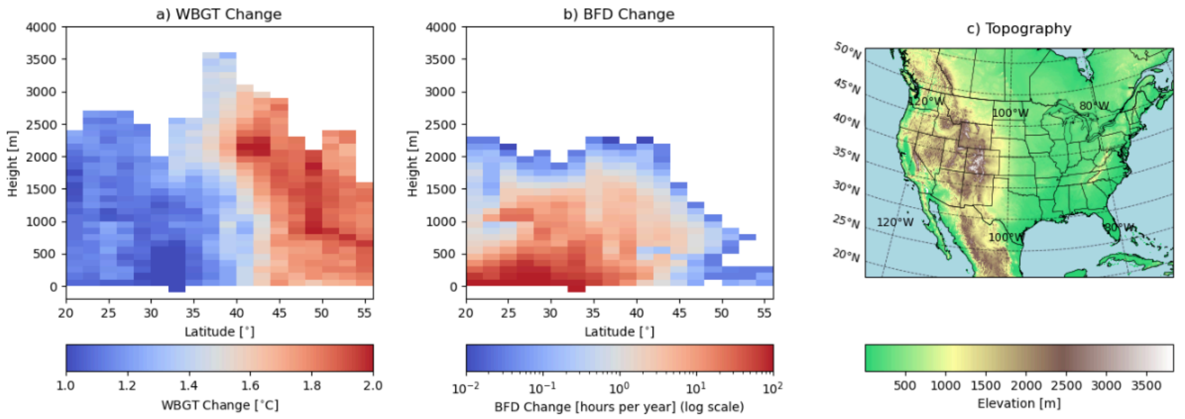

New Fig. S4: Annual maxima WBGT changes are primarily a function of latitude, while changes in BFD exceedances are more dependent on elevation. Annual maxima WBGT (a) and BFD exceedances (b) dependent on latitude and elevation at 2 °C of global warming (only land cells are included). The CONUS404 digital elevation model is shown in (c).

• I understand that the definitions of flag hours (Green, Yellow, and Black) are based on maximum WBGT values, which generally occur during the hottest hours of the day. However, in this study, the same flag hour thresholds appear to be applied to nighttime WBGT values as well. This raises an important question: Should a different set of thresholds be considered for nighttime conditions, given that human thermal physiology and exposure risks differ significantly between day and night? I understand that to partially address this issue, the authors used GFH instead of BFH. However, it would be valuable for the authors to clarify whether the use of daytime-derived thresholds for nighttime WBGT is methodologically appropriate and to discuss the potential implications of this approach for interpreting heat stress during nocturnal hours.

Thank you for raising this important point. Current WBGT-based heat-stress guidelines (e.g., ISO 7243 and U.S. military doctrine) do not provide separate daytime and nighttime thresholds. Instead, they apply the same WBGT limits whenever similar workloads occur, regardless of time of day. For this reason, and because no physiologically validated nocturnal-specific WBGT thresholds exist, most studies assessing nighttime heat stress use the standard WBGT categories to maintain comparability with established guidance.

At the same time, we agree that nighttime heat stress has distinct physiological implications, especially through reduced opportunities for thermoregulatory recovery and sleep. High minimum temperatures and “hot nights” are well known to increase sleep disruption and heat-related morbidity (e.g., Obradovich et al. 2017). Consequently, nighttime exceedances of standard WBGT flag thresholds should not be interpreted as equivalent to daytime work-rest recommendations, but rather as a relative indicator of extreme nocturnal conditions and impaired recovery capacity.

We added the following text to the revised manuscript to make readers aware of this point: L52-5: “**WBGT flag categories are defined for occupational heat stress and are not time-of-day specific. Warm nights are known to impair sleep and increase health risks even at lower heat stress. We account for this by analyzing exceedance of GFHs during nights recognizing that developing nighttime-specific WBGT thresholds remains an area for future work.**”

3. Interpretation of results

• The results of the study can be interpreted from two important perspectives: (1) the increase in maximum annual hourly WBGT (including nighttime WBGT), and (2) the exposure to elevated WBGT values over time. While the authors emphasize that the magnitude of WBGT increase is more pronounced in the northern U.S. under +1°C and +2°C PGW scenarios (Fig. 1 and Fig. S1), an equally critical insight lies in the spatial pattern of exposure to high WBGT values, particularly in the Eastern U.S. and Gulf of Mexico region. In these regions, WBGT values are already approaching or exceeding critical thresholds, and even relatively small increases under warming scenarios could have disproportionately severe impacts. This aspect of high baseline exposure compounded with warming deserves more attention and could be highlighted as an important finding of the study.

Thank you for raising this topic of baseline exposure. We added brief discussions of this result to the abstract, the results section, and the conclusion.

L21-24: Annual maximum WBGT increases the fastest in high-latitude areas, while heat stress increases the most in humid subtropical regions such as the eastern U.S., Gulf Coast, and Caribbean, where already high baseline WBGT amplifies exposure even under modest additional warming.

L119-21: While absolute increases in annual maximum WBGT are the largest in northern regions, the societal relevance of these changes depends strongly on background conditions, as regions in the eastern U.S. and along the Gulf Coast already operate close to critical WBGT thresholds.

L129-32: Notably, the strongest increases in WBGT exceedance occur in regions with already high baseline heat stress, particularly the eastern U.S. and the Gulf of Mexico region (Fig. 2). In these areas, even relatively small additional warming leads to disproportionately large increases in exposure because WBGT values are already near or above critical thresholds.

L254-6: This highlights that heat-risk amplification is not driven solely by the magnitude of WBGT change, but critically by pre-existing exposure levels, making humid subtropical regions especially vulnerable even under modest additional warming.

• The increase in nighttime WBGT exposure and its implications for Flag Hour categories is particularly important. This is a significant result with implications for adaptation planning. Given its relevance, it is surprising that this finding is presented only in the supplementary material. I recommend that this be brought into the main text and more prominently discussed.

Thank you for this comment. We decided to move the nocturnal GFH maps based on the CONUS404 simulations into the main manuscript (Fig. 2). To accommodate this, we moved the ERA5-based results to the supplement and mentioned that they agree well with those from CONUS404. We added additional discussion on the increasing frequency of nocturnal heat stress in the revised paper:

L139-146: **The annual maximum daily minimum nighttime WBGT (i.e., the hottest night each year; night being local 20:00–06:00) is increasing faster than the dailymaximumWBGT (Fig. S3). Nighttime GFHs were rare over land regions during the baseline period and have already increased in the aforementioned heat-prone areas at 1 ◦C global warming (Fig. 2e). A substantially larger increase is simulated under 2 ◦C of global warming, particularly in the eastern U.S. and Caribbean regions (Fig. 2h). Especially the heavily populated Gulf and Atlantic Coasts, as well as the southern Mississippi Valley, will see hundreds of hours more nocturnal GFHs per year than under current conditions.**

• In Fig S6, it would be interesting to know the locations of empirical exceedance probability at 1, baseline+1 (or 1.3) and 2 degree PGW. This will help in visualising the goodness of fit for the right-tailed exponential fit model.

We added the 1 °C global warming data point to the graphic in the revised manuscript. Unfortunately, we cannot add data points beyond 2 °C warming since the CONUS404 simulations have too few years at higher warming targets.

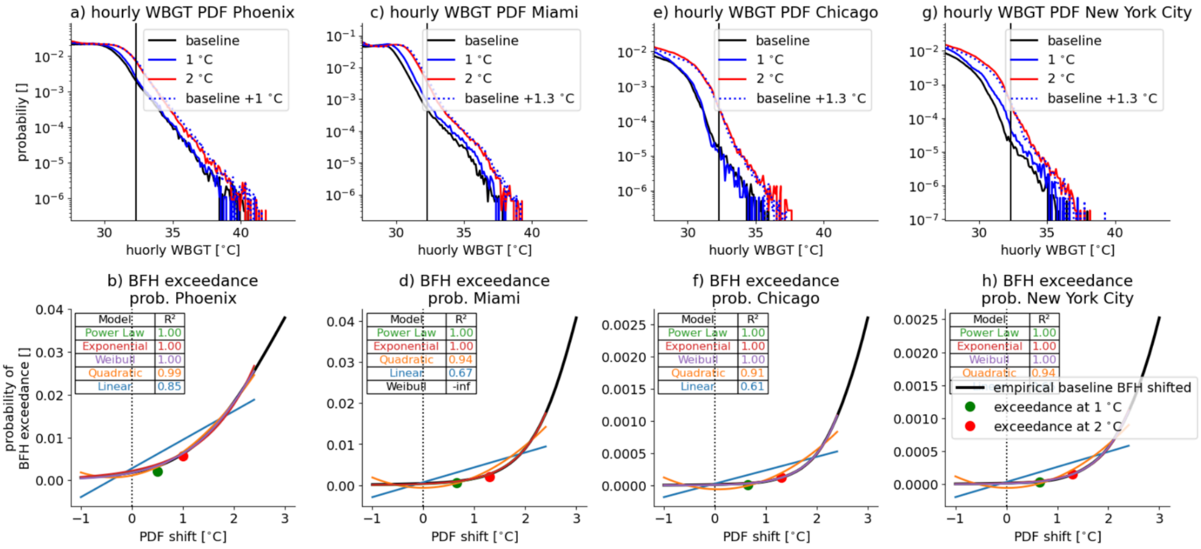

Fig. S8: The change in the hourly WBGT probability density function (PDF) with global warming can be very well approximated by rightward shifting the baseline PDF. The black flag hour (BFH) exceedance probability, thereby, increases approximately exponentially between -1 °C – 2 °C local warming. Hourly WBGT PDFs for Phoenix (a), Miami (c), Chicago (e), and New York City (g) under the baseline (black), 1 °C (blue), and 2 °C (red) global warming. The blue-dotted PFD is the baseline PDF shifted by 1 °C local warming in Phoenix and 1.3 °C in the other cities. The lower row shows BFH exceedance probabilities (black line) when shifting the baseline PDF to colder or warmer conditions. Different colored lines show the best fit of various functions to this data (colors correspond to font colors in the legend) between -1 °C – 2 °C. The coefficient of determination (R2) is shown for each functional fit in the inset table (higher is better). The green/red dot shows the empirical BFH exceedance probability at 1 °C/2 °C global warming derived from the CONUS404 CTR/PGW data.

• Population exposure to BFH for different US regions is also an interesting finding. I am curious to know whether we have the same pattern for the nighttime WBGT (even if it is for the population exposure to GFHs).

We added a similar figure to Fig. 3 to the supplement (Fig. S7, also see below) that shows population exposure of nocturnal GFHs. Similar patterns as for BFH emerge with an exponential increase in population exposure with warming. Regions in the eastern CONUS have roughly an order of magnitude more exposure to nocturnal GFHs compared to BFHs. We added a discussion of these results to the revised manuscript.

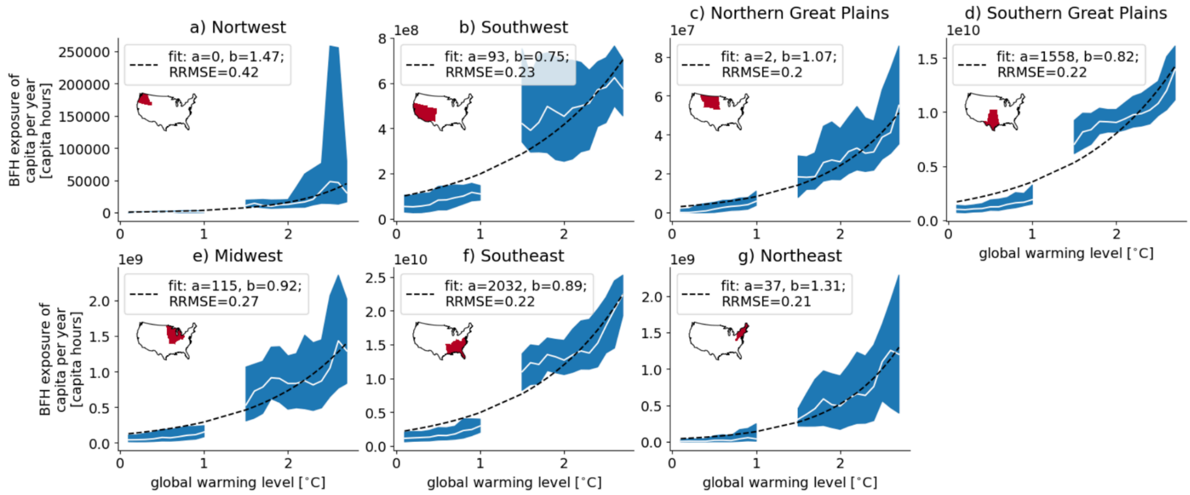

Fig. S7: The population exposure to nocturnal GFHs is increasing exponentially with advanced global warming with the largest relative increases in northern regions. The white line shows the median population exposure, and the blue polygon shows the interquartile spread from interannual variability (global warming levels are calculated by sampling over years within a window of ±0.25 °C). The red polygons in the inlet maps show the seven analysis regions. The dashed line shows the fit of an exponential function ($y = a \cdot e^{bx}$) where a is the initial value at no warming and b is the growth rate. The legend shows the best fit for a and b as well as the relative root mean squared error (RRMSE) of the fit.

4. Drivers of heat stress

The identification of prominent land drivers across different geographical regions of the U.S. is a particularly interesting part of the discussion. This aspect is important, as the characteristics and drivers of heatwaves can vary significantly across climatological zones. I agree with the authors' conclusion that heat mitigation strategies should be region-specific and tailored to the dominant local drivers of heat stress. I found the discussion in lines 154–182 particularly engaging. However, I have concerns regarding the conceptual framework used to support these insights-specifically, the WBGT sensitivity assessment based on the formulation from Kong and Huber (2025), as presented in Equation 1. This framework appears to assume that there is no collinearity among the meteorological variables (e.g., temperature, humidity, radiation, wind speed), which may not hold true in practice. How do the authors account for the potential influence of multicollinearity on the sensitivity coefficients? Without addressing this, there is a risk that the derived sensitivities may be distorted, leading to misinterpretation of the relative importance of different drivers. A brief discussion on this methodological limitation and any steps taken to mitigate its impact would strengthen the analysis.

Thank you for raising this concern. We want to clarify that the Kong and Huber (2025) WBGT sensitivity framework is physically based and derived analytically from the heat and mass transfer equations underlying Liljegren's WBGT model. The sensitivity coefficients in equation 1 are therefore not estimated empirically through multivariate linear regression, where multicolinearity among meteorological variables could affect coefficient estimates. Instead, these coefficients have explicit physical formulations.

These sensitivity coefficients are state-dependent (WBGT is a nonlinear function of temperature, humidity, wind, and radiation). To facilitate interpretation, we linearize equation 1 by evaluating these coefficients at each grid cell's climatology, conditional on the occurrence of annual maximum WBGT. This yields grid-specific, constant sensitivity coefficients, which are then used to diagnose the relative contribution of changes in each meteorological variable to WBGT changes at each grid cell. We also demonstrate the accuracy of this linearized sensitivity framework: summing up the relative contribution of each term can accurately reproduce model-simulated WBGT changes (Fig. S10).

However, we want to emphasize that this framework does not assume physical independence among changes in temperature, humidity, wind, and solar radiation. In reality, these variables are dynamically coupled. For example, wind and solar radiation changes can affect temperature and humidity through influencing monsoon circulation and surface energy balance. Therefore, equation 1 is intended as a diagnostic decomposition of WBGT changes into relative contributions from changes in individual meteorological variables. Specifically, it quantifies how changes in each variable affect heat and mass transfer between the WBGT sensors and the ambient environment, and thus the resulting WBGT values. It shouldn't be interpreted as a disentanglement of the underlying Earth system processes.

We have added relevant clarifications in the method section of the revised manuscript.

Literature:

Obradovich, N., Migliorini, R., Mednick, S.C. and Fowler, J.H., 2017. Nighttime temperature and human sleep loss in a changing climate. Science advances, 3(5), p.e1601555.

Reviewer #2 (Remarks to the Author):

This paper examines heat stress and its main drivers using Wet-Bulb Globe temperature (WBGT) as a measure to assess heat stress for human health. The measure is based on the combined effects of temperature, humidity, wind speed and radiation. Using factors in addition to temperature alone provides a better assessment of the true stress for the human body. The paper applies this measure to simulated climate scenarios to analyse WBGTs and threshold exceedance with global warming. Moreover, it identifies localized heat stress drivers. It does so using 80 year of historical and future climate data.

The paper finds that increases in yearly maximum WBGT are mostly found in high-latitude land regions. The frequency of threshold exceedance beyond 32 degrees WBGT (Black Flag Hours) are rising in humid subtropical regions. Moreover, once global warming of 2 degrees Celsius is reached and exceeded, the severity and frequency of heatwaves are likely to increase exponentially and also lead to heatwaves merging into a single prolonged heat wave, posing a high risk for human health and the economy.

Finally, the paper calls for mitigation and adaptation strategies and stresses that they need to be adapted to regional specificities for them to be most effective and efficient: in subtropical regions humidity is the main factor of concern; in arid high-latitude regions it is rather temperature increases and in other regions it is wind speeds that can amplify heat stress.

We thank the reviewer for their very constructive and insightful comments, which helped broaden the paper's perspective. While the revised paper focuses on physical processes, we added a range of new results that show how future warming will affect work capacity.

Comments and suggestions:

- The paper is very clear to understand, also for an economist, but we were expecting more analysis in terms of larger impacts such as socioeconomic outcomes in order for the paper to have more impact and go beyond a descriptive analysis.

- The paper's use of hourly data is a major strength, as this high temporal resolution is crucial for evaluating the effects of WGBT on socio-economic sector (e.g. human health and labour productivity). The dataset also offers strong spatial and temporal coverage, effectively capturing key phenomena such as the lack of nighttime cooling and the persistence of heat.

- To stress further the need for (tailored) adaptation and mitigation strategies the paper could add a section in which the economic risks of heat waves as well as the extreme scenarios if global warming reaches 2 degrees Celsius are explained in more detail. (see for example Current and projected regional economic impacts of heatwaves in Europe | Nature Communications which looks into effects on productivity). For example, even WBGTs at threshold levels below 32 degrees i.e. the black flag hours could already reduce productivity.

Thank you very much for raising these points. We agree that adding content on the potential economic impact of WBGT changes is valuable, while keeping the core focus of our study on the physical drivers of heat waves. We adopted the workability function from ISO/NIOSH used in Garcia et al. (2021) to assess potential changes in the working capacity during June, July, and August. We added corresponding images to Fig. 2 and Fig. 3.

We added panels c, f, and i to Fig. 2 and the corresponding discussion in the results section about changes in the June, July, and August work capacity under different warming levels. We also added panels e–h in Fig. 3 that show changes in work capacity as a function of global warming levels for different subregions.

- The dataset could be better exploited and possibly combined with the Inter-Sectoral Impact Model Intercomparison Project (ISIMIP) downscaled dataset to examine humid heatwaves' impact on socioeconomic outcomes.

We added the following statement to the conclusion section of the manuscript:
While our study is mostly focused on the physical drivers of increasing heat stress, future assessments could use our data for assessing heat stress impacts on economic outcomes and human and ecosystem health, for instance, within the ISIMIP (Inter-Sectoral Impact Model Intercomparison Project) framework.

- It would also be important to know what tailored mitigation and or adaptation strategies could look like. As the authors point out, they should differ across regions, with high temperatures mattering more in some regions and humidity more in others. Which tailored measures should be implemented? From an economic point of view, it would be interesting to know whether these measures would be costly or how they could be designed to be most cost-effective.

Thank you for raising this point. While this are very important questions, they go beyond what we can do in this article and would demand in-depth follow-on studies to assess adaptation strategies and their economics. We addressed this by adding the following context to our revised article:

L241-5: While our analysis highlights where temperature-, humidity-, or wind-related factors dominate rising heat stress, a detailed assessment of concrete adaptation options and their economic feasibility is beyond the scope of this work. Nonetheless, identifying the physical drivers provides a foundation for designing region-specific strategies, which future interdisciplinary studies can evaluate in terms of cost and effectiveness.

- Novelty: Regarding the main results depicted in Figure 4, we would like to know whether the authors agree that in a setting with 365 days per year the frequency of heatwaves must decrease with the length of heatwaves per year (with 10 heatwaves of 7 days, this implies already 70 days + at least one day below the BFH threshold to separate two heat events). Is the novelty of the finding that there is a turning point precisely at 7 heatwaves per year? And would this point be different under normal climate conditions? In case this is different, what could be the socioeconomic impact of this specific feature?

You are absolutely correct. With enough warming, we would reach the state of one continuous heatwave. The reason why this finding of merging heatwaves into long persistent heatwaves is important is that prolonged heat exposure has accumulative effects on human health and economic productivity. We added the following context to the revised article to emphasize this point:

L261-9: **Heatwave impacts on health and the economy depend not only on peak intensity but also strongly on duration and cumulative exposure.** Heatwave frequency and maximum annual duration increase linearly with warming until approximately 7 heatwaves per year are reached, beyond which continuous warming leads to the merging of heat events into prolonged mega-heatwaves, persisting for a month when global warming reaches 2 C in heat-prone regions. **Epidemiological studies show that mortality and morbidity rise over successive hot days due to accumulated physiological stress and limited nighttime recovery, with effects often persisting after the event[18,70]. Economic analyses likewise find nonlinear losses in labor productivity and output during prolonged heat, with damages scaling with the number of extreme-heat days rather than single-day peaks[71,72].**

- Methodology: how were the drivers of heatwaves identified? Are the drivers mutually independent?

Thank you for the question. We use the Kong and Huber (2025) WBGT sensitivity framework to decompose the relative contributions of changes in each meteorological variable (the drivers) to changes in WBGT extremes. The sensitivity framework (equation 1 in the method and copied below) is physically based and derived analytically from the heat and mass transfer equations underlying Liljegren's WBGT model. The sensitivity coefficients in equation 1 are therefore not estimated empirically through multivariate linear regression, but have explicit physical formulations.

$$\begin{aligned}\Delta WBGT &= \gamma_T \Delta Temperature + \gamma_Q \Delta Humidity + \gamma_{wind} \Delta Wind \\ &+ \gamma_{SR} \Delta Solar\ Radiation + \gamma_{P_s} \Delta Surface\ Pressure\end{aligned} \tag{1}$$

We then use equation 1 to diagnose the relative contribution of changes in each meteorological variable to changes in WBGT extremes at each grid cell.

However, we want to emphasize that this framework does not assume physical independence among changes in temperature, humidity, wind, and solar radiation. In reality, these variables are dynamically coupled. For example, wind and solar radiation changes can affect temperature and humidity through influencing monsoon circulation and surface energy balance. Therefore, equation 1 is intended as a diagnostic decomposition of WBGT changes into relative contributions from changes in individual meteorological variables. Specifically, it quantifies how changes in each variable affect heat and mass transfer between the WBGT sensors and the ambient environment, and thus the resulting WBGT values. It shouldn't be interpreted as a disentanglement of the underlying Earth system processes

We have added relevant clarifications in the revised manuscript (Line 628-36).

- It is important to explicitly mention both in the text and in the figures that point of comparison for the 1.5 degree or 2 degree warming is 1940-1969 instead of the Paris Agreement's time period 1840.

Thank you for raising this point. We added the following statement to the manuscript to address it:

L95-8: All global warming levels in this paper are relative to 1940-1969**, which is not the pre-industrial reference period defined in the Paris Agreement. Global mean temperatures during 1940-1969 were approximately 0.2-0.4 C warmer than mid-19th century pre-industrial conditions, depending on the dataset.**

Minor comments:

- Line 20: define explicitly BFH

We replaced BFH with heat stress in the abstract to keep it general.

- Line 81: remove "hourly"

Removed.

- Line 189: "black flag hours" instead of "black flack hours"

Fixed. Thanks for catching this!

- Figure 4: y axis should state [days]^-1 which is the proper unit used in physics for frequency (number of events per time period)

Thank you for pointing this out. The y-axis unit should be events year^{-1}. We changed the figure accordingly.

- Line 120: 273% increase of population exposure

Corrected.

- Please mask the oceans in all the graphs – very difficult to read otherwise

We thank the Reviewer for this suggestion. In this study, we intentionally retain the ocean areas in these figures to explicitly illustrate land–sea contrasts in the heat-stress response, which are an important aspect of the spatial patterns.

- Please review the colour scales that have continue colours in separate discrete number of colours, this will help readability of the charts. This is not for all the colour scales but for a few of them.

We revised all figures in the manuscript from continuous to discrete colors.

REVIEWERS' COMMENTS

Reviewer #1 (Remarks to the Author):

I thank the Editor for providing me with the opportunity to revisit the authors' responses to the comments I raised during the first review of the manuscript. I find that the authors have made a sincere and commendable effort to address the concerns. In doing so, they have undertaken additional analyses and incorporated further discussion into the revised manuscript.
Most of the additional analyses are satisfactory and strengthen the robustness of the findings. Overall, I am satisfied with the responses to my comments and with the additional work carried out by the authors.

Thank you for assessing our revised manuscript. We are pleased that the revisions addressed your comments.

My broad responses to the individual points are provided below.

Point 1:
I agree that in the absence of fine temporal resolution data, bias correction is difficult. I commend the authors to do additional analysis of comparing model simulation with observed data at daily scale. am satisfied by the response. The additional analysis that authors did is satisfying.

We thank the reviewer for the positive assessment. We are pleased that you found our response satisfactory.

Point 2:
I agree with the authors that there are no physiologically grounded or empirically validated WBGT thresholds. I am satisfied with the additional clarification provided in lines 197–210. The expanded discussion effectively underscores the importance of considering local and regional thresholds when drawing inferences about WBGT exposure. I appreciate this addition and find the revised discussion satisfactory.

Thank you for your comment and for raising this point.

Point 3 and Point 4- I am satisfied with the response and the clarification.

We are pleased that our replies addressed your concerns.
